# Biomedical applications of three‐dimensional bioprinted craniofacial tissue engineering

**DOI:** 10.1002/btm2.10333

**Published:** 2022-05-10

**Authors:** Nitin Bharat Charbe, Murtaza Tambuwala, Sushesh Srivatsa Palakurthi, Amol Warokar, Altijana Hromić‐Jahjefendić, Hamid Bakshi, Flavia Zacconi, Vijay Mishra, Saurabh Khadse, Alaa A. Aljabali, Mohamed El‐Tanani, Ãngel Serrano‐Aroca, Srinath Palakurthi

**Affiliations:** ^1^ Irma Lerma Rangel College of Pharmacy Texas A&M Health Science Center Kingsville Texas USA; ^2^ School of Pharmacy and Pharmaceutical Science Ulster University Coleraine UK; ^3^ Department of Pharmacy Dadasaheb Balpande College of Pharmacy Nagpur India; ^4^ Department of Genetics and Bioengineering, Faculty of Engineering and Natural Sciences International University of Sarajevo Sarajevo Bosnia and Herzegovina; ^5^ Departamento de Quimica Orgánica, Facultad de Química y de Farmacia Pontificia Universidad Católica de Chile Santiago Chile; ^6^ Institute for Biological and Medical Engineering, Schools of Engineering, Medicine and Biological Sciences Pontificia Universidad Católica de Chile Santiago Chile; ^7^ School of Pharmaceutical Sciences Lovely Professional University Phagwara India; ^8^ Department of Pharmaceutical Chemistry R.C. Patel Institute of Pharmaceutical Education and Research Dhule India; ^9^ Faculty of Pharmacy, Department of Pharmaceutical Sciences Yarmouk University Irbid Jordan; ^10^ Pharmacological and Diagnostic Research Centre, Faculty of Pharmacy Al‐Ahliyya Amman University Amman Jordan; ^11^ Biomaterials and Bioengineering Lab Translational Research Centre San Alberto Magno Catholic University of Valencia San Vicente Mártir Valencia Spain

**Keywords:** 3D bioprinting, bioengineering, biomaterials, craniofacial tissue complex, soft tissues

## Abstract

Anatomical complications of the craniofacial regions often present considerable challenges to the surgical repair or replacement of the damaged tissues. Surgical repair has its own set of limitations, including scarcity of the donor tissues, immune rejection, use of immune suppressors followed by the surgery, and restriction in restoring the natural aesthetic appeal. Rapid advancement in the field of biomaterials, cell biology, and engineering has helped scientists to create cellularized skeletal muscle‐like structures. However, the existing method still has limitations in building large, highly vascular tissue with clinical application. With the advance in the three‐dimensional (3D) bioprinting technique, scientists and clinicians now can produce the functional implants of skeletal muscles and bones that are more patient‐specific with the perfect match to the architecture of their craniofacial defects. Craniofacial tissue regeneration using 3D bioprinting can manage and eliminate the restrictions of the surgical transplant from the donor site. The concept of creating the new functional tissue, exactly mimicking the anatomical and physiological function of the damaged tissue, looks highly attractive. This is crucial to reduce the donor site morbidity and retain the esthetics. 3D bioprinting can integrate all three essential components of tissue engineering, that is, rehabilitation, reconstruction, and regeneration of the lost craniofacial tissues. Such integration essentially helps to develop the patient‐specific treatment plans and damage site‐driven creation of the functional implants for the craniofacial defects. This article is the bird's eye view on the latest development and application of 3D bioprinting in the regeneration of the skeletal muscle tissues and their application in restoring the functional abilities of the damaged craniofacial tissue. We also discussed current challenges in craniofacial bone vascularization and gave our view on the future direction, including establishing the interactions between tissue‐engineered skeletal muscle and the peripheral nervous system.

## 1 INTRODUCTION

The terms regenerative medicine and tissue engineering are often used interchangeably in medicine. However, regenerative medicine is a general term that incorporates tissue engineering and, at the same time, is concerned about the research in self‐healing. During self‐healing, the body uses its cellular mechanisms and foreign materials to recreate the cell and its functions and reorganize them in tissues and organs.^1^ Tissue engineering, on the other hand, has grown independently from the field of biomaterials. It combines the extracellular matrix (ECM) scaffolds, cells, and physiologically active compounds into functional tissue capable of improving or replacing the damaged one. FDA has already approved the engineered skin and cartilage in clinical use for limited indications. The field of tissue engineering is evolving rapidly, and its application is extended from the replacement of damaged tissues to the research tools to study the pharmacological activity, pharmacokinetics, educational models, etc.^2^, ^3^

Before the tissue regeneration and engineering concept, clinical options available to tackle the issue of tissue degeneration or loss of it were limited to organ transplantation, use of prostheses and implants, and transplantation of autologous tissue. Scarcity of organ donors, biocompatibility, and limited supply of autologous tissues (if tissue loss is more, e.g., skin burn) are some of the significant limitations of these approaches. Surgical reconstructions using autologous tissue along with the implants and prostate still have a widespread application when it comes to replacing volume or structural deficits.^4^ Complete replacement of the metabolic deficiencies using the surgical reconstructions approach is still an unmet challenge. Autologous tissue transfers have the additional problem of the other surgical site, with the risk of complications and donor site morbidity.

To some extent, organ transplantation has overcome some of the issues of autologous transplant. Organ transplantation can successfully replace the lost or damaged tissue and restore its physiological and metabolic functions. Whole organ transplants like liver and kidney have saved the life of several critically ill patients by restoring their vital functions.^5^ However, this approach has inherent limitations, including organ rejection and immunogenic risk, limited availability of donors, and regulatory approvals. Lastly, in the last few decades, prosthetics and implants have become highly advanced and sophisticated but are still limited in their use in replenishing the lost tissue volume or metabolic functions. Immune activation and distortion are still significant challenges that need to be overcome to optimize prosthetics and implants.^6^

At the organizational level, the tissue is placed between the cellular and organ level. A group of cells produces the necessary biochemical components and maintains physiological functionality. A particular group of cells also secrete the ECM/scaffold, which supports the structure and cellular growth and helps to transmit the signaling biomolecules essential for the organ's physiological function. In general, the local environment influences the role of individual cells. A group of cells can start the chain of actions via primary and secondary cellular signaling that determines the response of the same group of cells and surrounding cells. By understanding the primary and secondary cellular signaling processes and their effect on the individual and group of cells, the scientist is now equipped with sufficient advanced tools to manage and fix the damaged tissues or create new ones. The development of the new tissue or organs begins with the construction of the scaffold made up of biocompatible materials.^3^ Scaffolds were then seeded with the different types of cells supplemented with the growth factors. Tissue starts developing if the growth factors, cells, and scaffold are sufficient to provide the right environment. Alternatively, scaffold material, cells, and growth factors could be mixed, letting the tissue assemble and grow independently. One recent advancement in tissue engineering is decellularized donated organs and populating the remaining collagen scaffold with the cells to build new tissue. Ott et al. decellularized rat heart to get the myocardial scaffold, which was later repopulated with the myocardial and endothelial cells to revitalize the heart functions.^7^ These findings were extrapolated to the pig heart, confirming the scalability to the bigger organs.^8^ Wang et al., on the other hand, focused their attention on making the cardiac patch from the decellularized porcine heart.^9^ Studies are now emerged reporting acellular human myocardial scaffolds.^10^ Wang et al. also developed a robust protocol to decellularized the porcine heart to obtain the three‐dimensional (3D) acellular scaffold with very well preserved cellular gaps and ECM.^9^

Several studies confirmed the effectiveness of embryonic stem cells and adult mesenchymal stem cells (bone marrow or cord‐derived) in regenerating various tissues and organs; however, the long‐term viability of such reconstructs is limited due to the limited ability of cell division.^11^ To overcome the hurdle of limited cell division capability, the concept of multipotent progenitors derived from embryonic stem cells (ESC) is getting more popular because of their multipotent nature and proliferativeness, which enables them to recellularized the complex scaffolds.^12^ In the recent time, cells like pluripotent human embryonic stem cells (hESCs) have appeared as an attractive candidate stem cell source for obtaining complex tissues (e.g., cardiac cells) because of their remarkable capability for expansion and undisputed potential to differentiate into smooth muscle and endothelial cells including terminally differentiated cardiomyocytes.^13^ Identification of the multipotent stem cells, including the induced pluripotent stem cells, has raised new hope in the tissue engineering of complex tissue. However, the issues like nutrient and oxygen transport in thicker tissues, cell penetration, and toxicity of the degraded products of the scaffold are the major hurdles in its successful clinical application.^14^

Although remarkable development has been made in this field, engineered and regenerated tissue has some challenges that must be overcome before their clinical applications, including selecting appropriate cells, biocompatible scaffold, growth factors, low engraftment rate, and durability. To overcome the significant issues, alternative approaches for tissue engineering have emerged during the last decade. 3D bioprinting, which was initially developed for industrial purposes, is the latest approach adapted for tissue engineering, in which cells of interest in bioinks patterned in the desired shapes. The overall bioprinting process is controlled by the programs monitored by the computing systems.

The most critical factor in tissue engineering is the 3D scaffold, which provides a suitable microenvironment for cell proliferation and metabolic functions. A biocompatible material, stem cells, growth factors, and various imaging techniques have significantly supported the advancement in the field. Interdisciplinary research efforts from several areas have contributed immensely to developing the two‐dimensional (2D) flat, non‐vascular, and tubular organs that are being tested in the preclinical stage, and few are commercially available. On the other hand, solid, more complex tissues, including thick tissues, heart, kidney, and lungs, require innervation and vasculature to support oxygen and nutrient transport. This makes solid‐organ engineering much more complicated than flat 2D tissues. Solid 3D organs required more than one cell type with a 3D porous scaffold to support cell division and provide mechanical strength—this requires radial technical advancement to support the vessel growth within the 3D construct. One of the significant challenges in mimicking the natural tissues and organs is accommodating the multiple cell types and their spatial arrangement in 3D‐oriented ECM. Overall, the scaffold must be porous, biocompatible, biodegradable, or bioabsorbable for optimum growth and must provide mechanical support to the organ.

**FIGURE 1:**
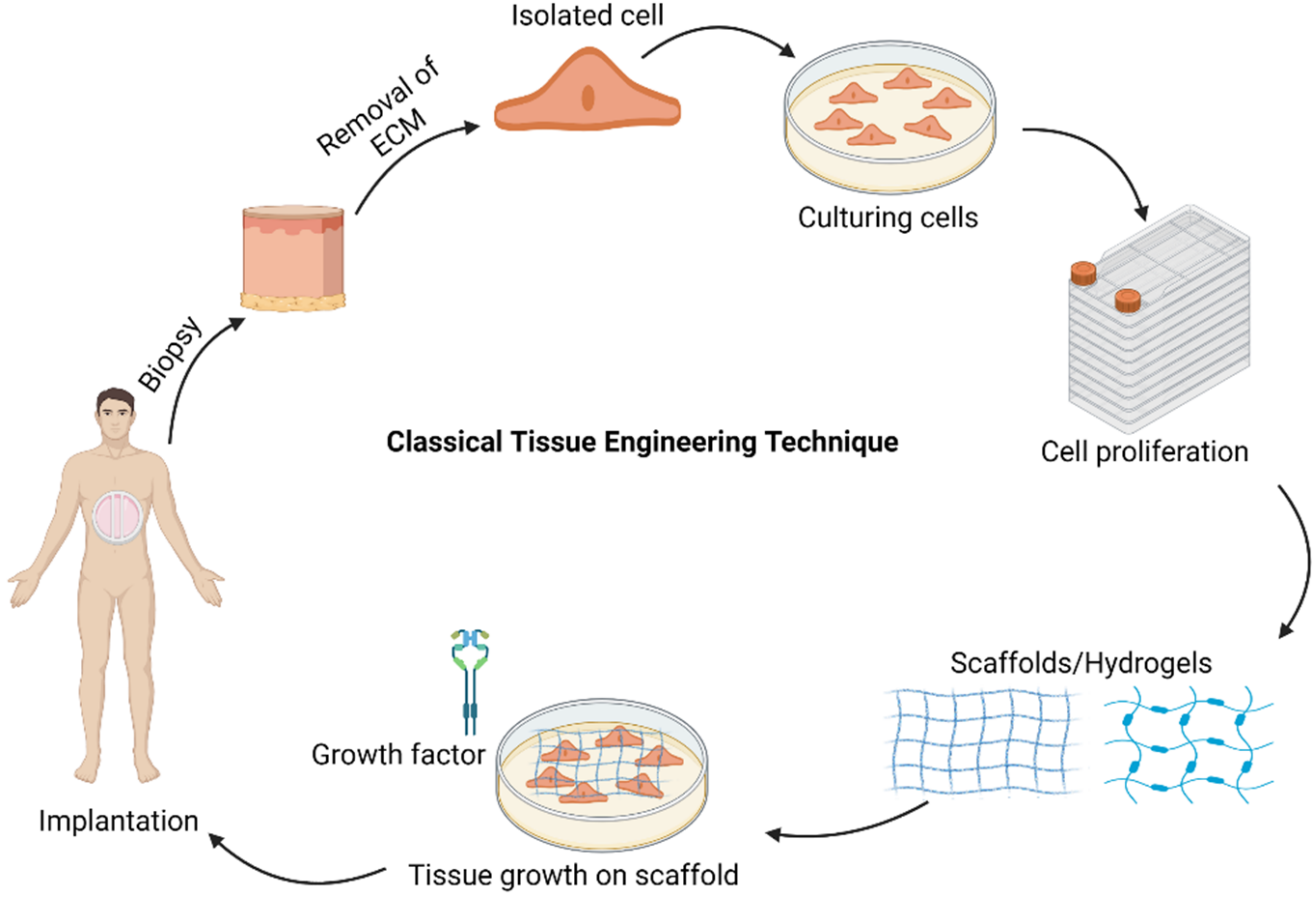
The classical tissue engineering approach

3D bioprinting offers precise control over the placement and layering of the cells within the scaffold. Compared to the traditional bioengineering method (Figure 1), 3D bioprinting allows higher precision in the space orientation relationship between the constituent elements of the tissue. In the near future, 3D bioprinting has the potential to overcome all the major issues of traditional tissue engineering. This review is the birds‐eye view on the advances made in 3D printing and its application in tissue engineering of bones and skeletal muscles. We also propose challenges and future viewpoints in implementing the principles of 3D printing and general tissue engineering to the craniofacial bones and skeletal muscles.

## 2 SOFT FACIAL TISSUES

In anatomy, at the organizational level, tissues are the structure between cells and the complete organ. A functional tissue is a complex of similar cells and ECM secreted by the participating cells. Tissues combine together to form the physiologically active organ—different tissues combine to serve a common function of the organs. In Vertebrate, tissues are grouped into connective, muscle, nervous, and epithelial tissues.^15^ The appearance of all the tissues varies depending on the type of the organism, organs, and precursor cells. For example, during embryonic development, endoderm and ectoderm give rise to the epithelium layers. A minor contribution from the mesoderm gives rise to the specialized epithelium that creates the vasculature.^16^ A typical epithelial tissue is covered by a single layer of cells with tight junctions having selective permeability.^17^ Epithelial tissues cover all the tissue surfaces that come in direct contact with the external environment, such as the digestive system, oral cavity, breathing tracks, and skin. Its location explains its functions of selective absorption, secretion, protection, and separation from the adjoining organs.^17^ Similarly, neural ectoderm gives rise to neural tissues like the brain, motor nerves, retina, etc., and non‐neural ectoderm gives rise to epidermal tissues like nails, hair, feathers, breaks. On the other hand, mesoderm gives rise to skeletal tissues (e.g., bone and cartilage of skull), connective tissue (e.g., dermis, fat), muscular tissues (e.g., voluntary muscles), and vascular and hemato tissues (e.g., vessels, osteoclasts).^18^ Whereas endoderm developed to form pharyngeal tissue (e.g., Auditory tube) and glandular tissues like thyroid, thymus, parathyroids. During embryonic development, the neural crest also gives rise to skeletal tissues (bone and cartilage, dentin), neural tissues (Neurons, sensory ganglia, glia), connective tissues (dermis, fat), and vascular tissues (pericytes, smooth muscles).^17^

The skeletal muscles of the head are known as craniofacial muscles.^19^ Superficial epidermis and dermis layers are innervated with nerves by the peripheral nervous system. A little deeper, the muscles that control the expression (smile, wink, superficial muscles of expression) are placed. Deeper, craniofacial complex has the muscle of chewing (pterygoid, temporalis, masseter, digastric, mylohyoid, etc.), which helps close or open the jaws. The craniofacial complex also has the eyes' muscles that move them in orbit, for example, extrinsic ocular muscles.^19^ Blood vessels that bring oxygenated blood and remove deoxygenated blood (part of the circulatory system) are the crucial component of the complex. Different tissues like skeletal, vascular, muscular, nervous, and connective tissue contribute to the head complex.^20^ During embryonic development, the neural crest gives rise to skeletal tissues (bone and cartilage, dentin), neural tissues (Neurons, sensory ganglia, glia), connective tissues (dermis, fat), and vascular tissues (pericytes, smooth muscles). All these tissues do not form in isolation but rather interact with each other to form so that each cell knows where to connect to blood vessels and bones.^20^

In parallel to tissue engineering, the present research is also focused on how all these muscles come together and form a complex system. The current research focuses on identifying the molecular and cellular mechanisms and processes that control head and craniofacial muscle development and the structural integration of all these parts to form the complex.^21^ How this muscle knows where to attach to bones and move in relation to other structure and how the blood vessels and connective tissues come together with hard and soft tissues to build craniofacial complex is also the unresolved mystery. For craniofacial tissue engineering, it is crucial to know how the precursor cells learn where and when to differentiate into appropriately patterned head components.

The craniofacial system is the most affected system in terms of congenital disabilities, somewhere around 1 out of 300–500 live birth.^22^ Study of the origin of this tissue, differentiation, and integration could be useful to regenerate these tissues in disease, congenital disabilities, or in cases of trauma.

## 3 LIMITATIONS OF CURRENT SURGICAL OPTIONS

One of the most devastating, frequent, and expensive problems in healthcare is the partial or complete functional loss of tissue or organs. The most widely used treatment option is surgical repair, mechanical devices, or tissue transplantation from a different site or the matching donor. Unlike modern tissue engineering techniques, which are still in their initial development phase, the classical surgical option is still preferred. Modern tissue engineering aims to replace the damaged tissues with implants or constructs that could maintain and restore the natural function of the damaged tissues or organs. Although artificial skin and cartilage are some of the engineered tissue that has been approved by the FDA, they still have limited application. Although surgeries are still one of the most widely used options to restore the function of the damaged tissues, they still have their inherent problems. On the other hand, tissue engineering is based on the principles of engineering, molecular biology, and material science to develop functional substitutes to replace, restore, and improve the lost functions of tissues and organs.

Surgical approaches evolved over time, including replacing the damaged tissue with the tissue of the unaffected site in the same individual and organ transplantation from the donor individual. Surgical strategies like replacing damaged tissues and organs with artificial devices such as joints, heart valves, and bones have evolved over time. Surgical procedures often are not enough to recapture the original physiological functions of the tissues and hence require supplements to support the lost metabolic function. This often required the use of growth hormones, calcium, proteins, etc. Surgeries are benefited by the significant advances in the field of medicines, but they have several limitations of their own, which includes (1) hormonal supplements are most widely used in case of the loss or damage of the endocrine glands, for example, insulin is chronically given to lose of pancreatic gland. Such hormonal replacement therapy to normalized the metabolic functions often leads to hormonal imbalance, for example, insulin imbalance could lead to the hypo glycemic or hyperglycemic situation and several other physiological complications. (2) Implants often require repeated surgeries; this is crucial in pediatric patients because, in such patients, they are required to replace because of the growth and anatomical changes. (3) The major issue is the lack of biocompatibility and immune rejection in the case of the implants made up of nonbiological materials like keen joints, mechanical valves for the heart, stents, prostheses, etc. (4) Implants made up of nonbiological materials sometimes lead to the complications like thrombosis, carcinogenicity, bacterial infections, and toxicity due to the degraded products. (5) Surgical replacement of the damaged organ or tissue with the different tissue type often lacks the ability to mimic the original function in a new environment. The donor site also suffers major damage in such procedures. Furthermore, increasingly donor scarcity also limits the surgical options. Another major limitation is the high cost and serious side effects of the immunosuppressant drugs, which are required for a long time. On the other hand, tissue engineering provides a novel solution to the replacement of damaged or lost tissues. The tissue engineering principles are based on replacing the lost function with the constructs or implants developed from the host's own cells or from the donor's cells. With the advancement in cell engineering and biomaterials, the day is not far when the living tissue developed and constructed in the lab will replace lost tissue and organ functions, eliminating donors' needs. In the modern tissue engineering approach, a healthy site of the patients could use to donate the cells, which can then expand in the lab, essentially eliminating the risk of immune rejection. Moreover, during in vitro development, if required, cells could be genetically modified to repair the genetic fault before implanting the developed genetically modified tissue on the required site. This approach not only eliminates the need for donors and the long waiting list for the patients, but surgeons can adapt with the advances in tissue engineering to carry out minimally invasive surgery. Tissue engineering is now an established technique, and promising results are coming worldwide. Hopes are very high; the need is massive, and potential benefits are endless. However, much work still needs to be done, and many questions remain unanswered. One of the most fundamental questions is the expansion of the target cells and the generation of the growth signals, which could direct the cells to form functional 3D organs with proper vasculature for oxygen and nutrient supply. Knowledge of the basic physiology of the tissue and individual cells is complimenting the growth of tissue engineering. However, signaling to govern the tissue growth and cell migration in culture and in vivo needs better understanding. For example, understanding why skeletal muscle satellite cells multiply rapidly while cardiac myocytes do not divide at all in culture is essential. Cardiomayocytes are terminally differentiated and cannot replace the damaged site due to the infraction. Understanding the signaling that governs the rapid division of the skeletal muscle satellite cells could be useful to modify cardiomyocytes to replace the infracted site genetically. Similarly, liver cells rapidly regenerate in vivo however grow poorly in culture. Remarkable progress has been made in identifying the organ‐specific stem cells and their ability to differentiate into required cells types. Stem cells hold the ability to provide a limitless supply of cells. However, it is necessary first to identify the protocol and standardize it to isolate the stem cells and confirm their ability to differentiate into required cell types. At the same time, a detailed investigation of the signaling and growth factors is required, which leads to differentiation. One major task is identifying and isolating the subpopulation and investigating their characteristic features that contribute to their division and chemotactic migration to form the specific organ. To overcome the challenges of tissue engineering, close collaboration between clinicians, biologist, chemist, material scientists, engineers are required. In addition, the adaption of 3D bioprinting, which has the highest feasibility toward the synthesis of living tissues, is essential to meet the inherent challenges of tissue engineering.

## 4 3D PRINTING: THE CURRENT STATE OF THE ART

3D printing, an additive manufacturing technique, is widely used for the precise fabrication of tissues. One essential requirement for the bioprinting of tissues and bones is the materials compatibility for the bioprinting process.^23^ 3D fabrication using printers requires printable biomaterials compatible with the live cells. Other crucial factors are non‐toxicity, crosslinking ability, biocompatibility, sufficient load‐bearing ability, shear‐thinning properties, and support for cell proliferation and adhesion with adequate plasticity.

Like noncraniofacial bones and tissues, the craniofacial complex is composed of nerves, blood vessels, bones, cartilages, muscles, and ligaments. Together, these complex components perform several face functions like speech, smile, mastication, and esthetics. Irreparable damage to the craniofacial complex could have a long‐term psychosocial impact highlighting the requirement of precise restructuring of the damaged part.^24^ For restructuring, if a transplant is required, the autologous source is considered a gold standard. However, in the case of significant damage, the autologous source could not be sufficient to fulfill the volume. This makes tissue engineering a potential source of bones and tissues for transplantation. As esthetics are an important feature, precession in craniofacial tissue engineering is crucial. In general, the craniofacial complex has several similarities with the other organs and tissues. Hence, the concepts of surgeries, therapies, tissue culture, transplantation, and 3D bioprinting are also applicable to the craniofacial complex. However, due to the complex geometry, craniofacial bones and tissues engineering face unique hurdles.

3D bioprinting involved fabricating the structure similar to the one that needs replacement by depositing biomaterials loaded with the cells (bioinks) or without cells (mostly for scaffold) at the micrometer scale. 3D printing takes place with the help of an extruder move along three axes oriented in space.^25^ The movement of the extruder along all the axes is controlled by design developed using an image program and shaved in file format (e.g., g.code) that is followed by the printer. Due to the potential of 3D printers in tissue engineering, their application has increased over the last few years, and various printable bioinks with printing properties like printability, flexibility, and printing fidelity have been developed.

In order to create a bioprinter that is capable of producing complex artificial tissues several innovations in extrusion, stereolithography, Inkjet and laser printing, is taking place. One of the most sought after latest advancement is the two photon polymerization based 3D bioprinting.

Two‐photon polymerization, which was first demonstrated by the Göppert‐Mayerin 1931, allows the fabrication of the 3D complex structures and critical dimensions of the order of 100 nm.^26^ Since then, it has found applications in microrobotics,^27^ biosensing,^28^ biomedical research,^29^ etc. The key functional elements of the two‐photon polymerization techniques are the lasers, which are able to provide the pulse of the femtoseconds, photosensitive material, stage and the program, and the computer to monitor the process of polymerization. It offers the huge 3D designing freedom by precise direct laser point polymerization. These traits are highly crucial for the 3D printing of tissues and bones.

Among the other 3D printing principles, two‐photon polymerization‐based 3D printing offers the best spatial resolution because of its nonlinear light‐induced effects in the photosensitive material. During the process of two‐photon polymerization, the oxygen present in the surrounding quenches the radicals up to some extent. This ultimately helps the process to take size down to around 100 nm. Another distinct advantage of this process is that many polymers have almost nonlinear absorption in the near infra‐red region of the spectrum, which help the laser to penetrate deep inside the material. This feature helps creating nano structures that are otherwise difficult to build.

**FIGURE 2:**
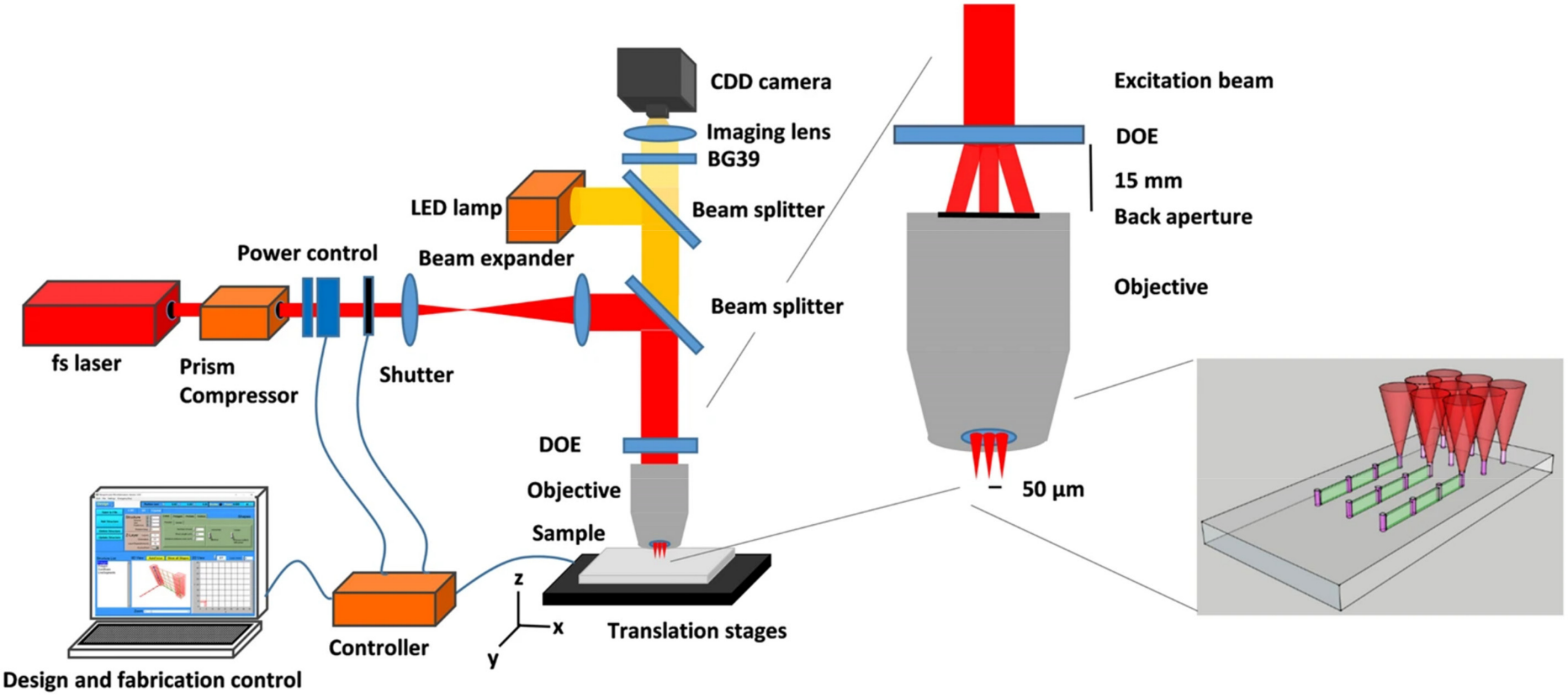
Schematic representation of the TPP experimental setup^30^

The first commercial two‐photon polymerization‐based 3D printer was made available by Nanoscribe in 2007. Other major commercial payers are Microlight3D, Multiphoton Optics, UpNano, and Femtika. 3D printing by two‐photon polymerization is a direct laser writing technique in which the solid structure is written into a liquid resin voxel‐by‐voxel, by scanning a femtosecond‐pulsed tightly focused laser beam (Figure 2). The commercial system typically uses the pulsed laser with a pulse repetition of the order of tens of MHz and the light in the range of color green and near infrared or the combination of both.^31^ The most common resin used with system is acrylate or epoxy derivatives. In the recent times as the application of the two‐photon polymerization‐based 3D printing expanded to the several field including the biomedical research, the development of the in‐house and more versatile material is dominating the field of material research.

As two‐photon polymerization application in biomedical research increased, research on the effect of the geometric topographies in the differentiation of the iPSC imitated by Aliasgar et al. The group printed the topological patterns of different spatial size and arrangement and studied their effect on the cellular differentiation. The group found out that when iPSC attached to such patterns for a week, the cells started expressing several genetic markers, which are the hallmark of differentiation of the stem cells toward the heterogeneous population of multipotent progenitors from all three germ layers.^32^ Similarly, Nieder et al. demonstrated that cell proliferation increases in the presence of the 3D microstructures compared to the planer surfaces.^30^ The group also confirmed that the cell adopted the elongated morphology when cell are attached to the 3D microstructure surface. Both observations indicate that the 3D microstructures fabricate using two‐photon polymerization could be the tool to study cellular interaction, cell signaling, migration, cancer metastasis, and tissue engineering.^30^

Overall, two‐photon polymerization‐based 3D bioprinting is an excellent approach for the development of the nanoscale structures. Its application in the biomedical and tissue engineering is not only limited to the fabrication of the 3D bioprinted tissues and drug delivery vehicles but now extended to the test the effects of the geometric topographies on the stem cell differentiation. In future, two‐photon polymerization could be the catalyst for the development of the self‐healing and self‐regenerating 3D tissues where stem cells could be patterned in the two photon polymerized nanoscale scaffold, which then depending on the spatial arrangement, and clues will differentiate into the required cell type.^32^

## 5 BIOINKS FOR 3D BIOPRINTING OF SOFT SKELETAL TISSUES AND BONES

Bioprinting is an excellent opportunity to engineer 3D tissues and organs that match and mimic anatomical and physiological functions. ECM, which governs many physiological functions of the cells apart from giving the structural features, is difficult to replicate artificially because of its complexity. The success of bioprinting depends on the survival and proliferation of cells in the constructs. In recent times to enhance cellular viability, several innovations have taken place in design and materials development (e,g biopolymer, hydrogels,). The formulation of live cells in biomaterial, which facilitates the task of bioprinting, is called bioink. They must meet certain characteristics like biocompatibility, physio‐chemical, and rheological properties to be effective. Bioink is considered the most advanced innovation in bioengineering as it provides higher reproducibility with accurate control over the anatomical features. At the same time, it offers flexibility and can be extruded out as filaments or droplets from the nozzles. Irrespective of the various advantages, its overall adaptability depends on how sensitive the biomaterials is to the bioprinting process.^33^

Fundamentally, bioinks must copy the functions of cell support, proliferation, differentiation, and cell‐ adhesion from ECM of the target tissue. Further, to be printable, bioinks should have the optimum rheological properties. Bioinks with think vicious constancy are mostly suitable for extrusion‐based bioprinting, whereas less viscous bioinks are suitable for inkjet bioprinting. The gelling time along with the viscosity of the bioinks determines the resolution of the fabricated construct.^34^ As high polymer in the bioink is not suitable for cell migration and proliferation, a recent trend is toward using less polymers in bioinks to support better cell growth.^35^ The development of suitable bioink is a dynamic area of research, especially for soft skeletal tissue engineering.

Most of the bioink characters match with the hydrogel. However, hydrogels intrinsically do not have the printable filament formation property. Hydrogels are often developed into pippeitable and filament‐forming forms, which could be cast into the molds. Transformation of the hydrogels into printable filament formation form is often done before printing or sometime after the deposition. Classically, bioprinting involves the continuous deposition of cell‐laden biomaterials onto the support. As higher shear stress could damage the cell and affect its viability, reducing it nozzle diameter and pressure need to be taken into account while printing. Overall the parameters that influence the printing process include (1) viscosity of the bioink, (2) temperature at the nozzle (to reduce the cell damage), (3) feasible crosslinking process, (4) uniformity in filament formation, (5) optimum pressure (to reduce the cell damage), and (6) gellation.^33^

**FIGURE 3:**
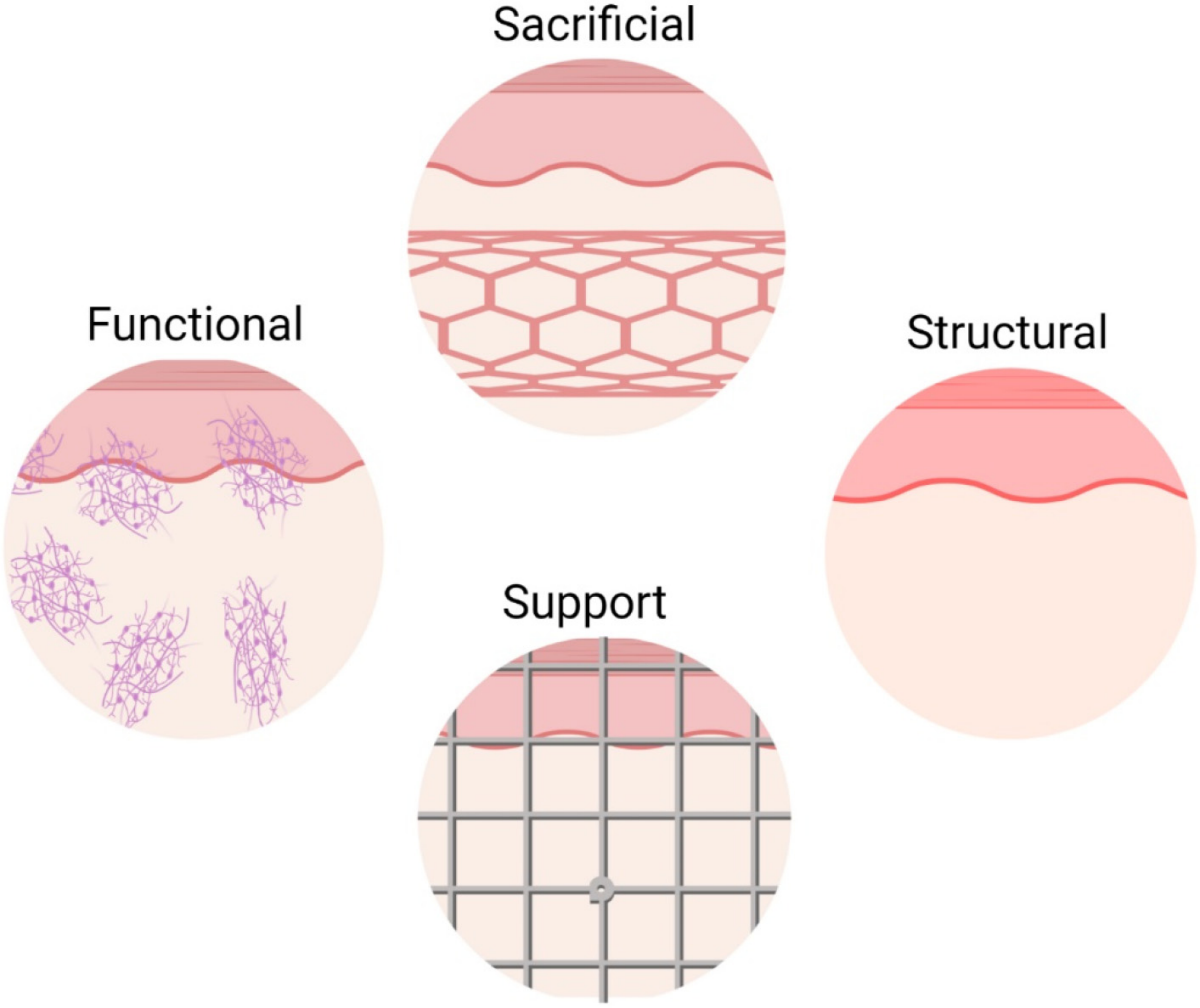
Different types of bioinks

Based on the functions, bioinks are classified into: *Structural bioink*: These bioinks are mainly used to create the frame of the structure. The most commonly used polymer/biomaterials to make this bioink included gelatins, alginate, cellulose, decellularized, and demineralized ECM, etc. The selection of materials for bioinks depends upon required properties, including cell viability, shape, and size.^36^ *Sacrificial bioink*: As the name indicates, these bioinks are generally removed from the fabricated structure to give rise to the desired geometry within the structure. Most widely, such bioinks are used to make channels to mimic the natural vasculature. To conveniently remove from the structure, the properties of such bioink need to be different from the surrounding material. These bioinks are crucial to make the thick, functional tissues and organs with proper arrangement for the transport of oxygen and nutrients. Carbohydrates, sugars, pluronic, and uncrossed gelatins are the most commonly used sacrificial materials.^37^ *Functional bioink*: These bioinks are very crucial for the success of the final constructs. They are not only associated with the structural integrity and functions of the constructs, but primarily it is associated with the differentiation of the cell. Other than the biomaterials like polymers, these inks often contain the growth factors to stimulate stem cell differentiation.^38^ *Support bioink*: as the name indicates, these bioinks are widely used to offer support to the final construct. These bioinks are meant to grow the construct up to the desired points, after which the construct supports themselves. These bioinks could also be removed from the fabricated structure once they start to support themselves (Figure 3).^39^

Synthetic and natural polymers are widely explored for their utility in the bioprinting of skeletal muscles. Natural polymers like fibrin, alginate, collagen, and gelatin calcium alginate have been used widely for skeletal muscle fabrication for better crosslinking and cell‐supportive properties.^40^ Among them, alginate is the most popular natural polymer used in modern 3D bioprinting because of its fast and reversible ionic gelation (in the presence of CaCl_2_). Tamayol et al. recently used alginate as a sacrificial bioinks to entrap and polymerized different polymers like gelatin, agarose, gelatin methacryloyl, polyethylene glycol diacrylate (PEGDA), and polyvinyl alcohol. The process involves two steps; in the first step, alginate is used to entrap various pre‐polymer solutions physically; in the second step, pre‐polymeric solution entrapped inside the alginate network is crosslinked to form an independent polymeric network.^41^ For personalized patient care, Negar Faramarzi et al. prepared the alginate‐based bioink using patients' own platelet‐rich plasma. Platelet‐rich plasma is believed to contain growth factors to induce angiogenesis, stem cell recruitment, and tissue regeneration.^42^

To enhance the strength of the structure, a group like methacryloyl was added to make various derivatives of synthetic and natural polymers. Methacryloyl derivatives of the polymers have better‐crosslinking characters. Few methacryloyl derivatives with the crosslinking character better than their natural counterparts are gelatin methacryloyl (GeMA), hyaluronic acid methacrylate (HAMA), carboxymethyl cellulose methacrylate (CMCMA), glycidyl methacrylate (GMHA), oxidized methacrylate alginate (OMA), and methacrylate alginate (MA).^43^ Photocorsslink is one of the most widely used crosslinked methods employed to crosslink these derivatives. UV spectrum of radiation is the most widely used source and its intensity and time of exposure determine the extent of crosslinking, cell viability, and mechanical strength. Due to the cancerous nature of the UV light, although less instance, the visible light source also offers the alternate approach to the UV light. Luiz E Bertassoni et al. developed a photolabile HEPG2 cell‐laden bioprinting method for bioink made up of methacrylate gelatin hydrogels.^44^ Cells like MCF‐7, NIH 3T3, and HUVECs were found to survive and perform well when presented in a bioink composed of 1.5%–2% gelatin methacryloyl hydrogels then 1% GelMA.^45^ Jia et al. to prepare vasculature smooth muscle used bioink composed of gelatin methacryloyl, sodium alginate, and 4‐arm poly(ethylene glycol)‐tetra‐acrylate bioprinted with two‐layered coaxial extrusion 3D bioprinting system.^46^ Endothelial cells and mesenchymal stem cells, which were present in the bioink, differentiated into the smooth muscle cells in the presence of TGF‐β1.^46^

Decellularized ECM (DeECM), which could be obtained by discarding the native cell to leave behind the ECM scaffold, is also explored as an option for bioink. As such materials are obtained from the tissue itself, it has the advantage of being close to the natural tissue and hence is considered as the best choice for tissue and organ regenerations. DeECM is also found to contain the cytokines, various proteins, and proteoglycans that could assist the stem cell in differentiation, proliferation, and adhesion.^47^ To date, various tissues are regenerated using the DeECM based bioinks; the most prominent among them are bone, spinal cord, brain tissue, vasculature, adipose tissue, heart, liver, kidney, and skeletal muscles.^48^ As DeECM‐based bioinks are weak with low‐stress bearing capacity, it requires a stiffening agent to carry out the crosslinking. To overcome this issue, DeECM‐based bioinks are prepared by mixing components like gelatin and PEG derivatives to enhance mechanical properties and viscosity.^49^

Synthetic polymers like PLGA, PCL, PVA, and PEG are also used widely for bioinks. Among them, PEG‐based hydrogels are used most popular.^50^ Further, to make them photocrosslinkable various methacrylate and acrylate derivatives of such polymers are prepared. Some of the examples of such derivatives are polyethylene glycol diacrylate, poly(ethylene glycol) dimethacrylate, poly(ethylene glycol‐co‐lactide) acrylate, and poly(ethylene oxide) dimethacrylate.^51^ Polyurethane, which is a synthetic derivative, is widely accepted for medical and tissue engineering purpose because of high stress‐bearing capacity, flexibility, and biocompatibility. Toxicity is one of the major concerns of synthetic polymers and their derivatives. To overcome this challenge, research is now focused on developing the less toxic variants of such polymers by using green chemistry and removing toxic ingredients from synthesis process.

Natural polymers can adhere better with cells as compared to synthetic polymers, whereas synthetic polymers offer unique strength and tuneability when compared with natural polymers. To get the best from both classes, composite material bioinks made of synthetic‐natural, natural‐natural, and synthetic‐synthetic polymers were made to get the ink with good cell adhesion and supportive cell properties. Such composite bioinks are close to the ideal parameters of the bioinks. Oju Jeon et al., in their recent work, used oxidized methacrylated alginate/8‐arm poly(ethylene glycol) amine to prepare cell‐laden bioink of human bone marrow‐derived mesenchymal stem cells. The group demonstrated that this composite bioink is tunable by varying the degree of alginate oxidation and shown a biocompatible character.^52^ Similarly, Luo et al. developed a 3D printed highly porous scaffold using a composite of highly concentrated alginate and polyvinyl alcohol.^53^

It is challenging to prepare a single biomaterial that could be used to make bioinks for different cells and purposes and satisfy all the requirements of tissue engineering and regeneration. To overcome the issue, García‐Lizarribar et al. developed the library of different composites to be suitable for different tissue engineering needs. Natural polymers like cellulose and alginate were derivatized using methacrylic anhydride, and synthetic polymers poly(ethylene glycol) diacrylate were mixed with gelatin methacryloyl to obtain photopolymerizable hydrogel composites.^54^

To form multiple microfilaments fibers or droplets, microfluidic heads were developed. The microfluidic head technique allows a fast switch between different bioinks to form the fibers of different bioinks.^35^ Costantini et al. developed a new 3D bioprinting method to construct artificial skeletal muscle tissues. The group combined two different cell‐laden (C2C12 and BALB/3T3 fibroblasts) bioinks made up of PEG‐fibrinogen/alginate using the microfluidic head. Myotube formation was noted on the side seeded with C2C12 cell‐laden bioink.^55^ Cameron J. Ferris et al. developed the specialized bioinks suitable for the drop‐on‐demand type of printing. To prepare the bioink Dubelcco's Modified Eagles Medium mixed with Poloxamer 188 was used to hydrate the gellan gum. The ink was found to be suitable for a drop of demand printing, with reproducibility and without cell precipitation.^56^

Stimulus responsive character was exploited for the development of the smart bioink. Polymer like poly(N‐isopropylacrylamide) has a low critical solution temperature of 32°C and allows the phase transition at 32°C from liquid to gel phase (above 32°C).^57^ This property enables the bioink to be in the liquid phase during the printing process. It turns to gel when it comes to the surface, having a temperature more than the critical solution temperature. This quick conversion from liquid to gel allows cell‐laden bioinks to maintain the shape of the bioprinted structure. Shear stress was also used to prepare the stimuli‐responsive smart bioink. Bioinks prepared from such materials loosed their viscosity under a high shear rate, allowing better printing under high pressure.^58^

Kim et al. too fabricated the human skeletal muscle prepared the cell‐laden functional and sacrificial bioink. Functional human primary muscle progenitor cells‐laden bioink was prepared using fibrinogen, gelatin, hyaluronic acid, glycerol. The sacrificial bioink to generate the vasculature for the muscle was prepared using gelatin, HA, and glycerol. The bioprinted skeletal muscle preparation using these bioinks has shown that the bundle of muscle was composed of a tightly packed myofiber‐like structure.^59^ Seyedmahmoud et al. prepared the hierarchical skeletal muscle to match the function of native tissues. They used C2C12 myoblasts cell‐laden gelatin methacryloyl (GelMA)‐alginate bioinks. The observations confirmed that the 10% (w/v) GelMA‐8% (w/v) alginate crosslinked using UV light and 0.1 M CaCl_2_ delivered the optimum condition to stimulate muscle tissue formation compared to other hydrogel compositions. Moreover, the improved metabolic function was seen with the addition of oxygen‐generating particles to the bioinks.^60^

### 5.1 Cell‐laden bioinks for craniofacial tissue regeneration

For craniofacial tissue engineering, no specializes bioink is reported in the literature; however, considering the similarity of craniofacial skeletal tissues with non‐craniofacial skeletal tissues, cell‐laden bioinks developed for the skeletal muscle are applicable for craniofacial tissue regeneration. Skeletal limb and trunk muscles, during embryogenesis, are originate from paraxialmesoderm known as somites. Craniofacial muscles, which control facial expression, vision, mastication, and esthetics, arise from cranial paraxial mesoderm of pharyngeal arches. The difference in the origin of the two is also complemented by the different control over the gene expression controlling their development. Somite‐derived myogenesis is controlled by transcription factors PAX3 and PAX7, whereas craniofacial myogenesis is regulated by the transcription factors PITX2, TCF21, and HC2. Myosin heavy chain (MyHC) part of the myosin is responsible for the contraction of the muscle by acting on the actin fiber.^61^ Craniofacial muscle expresses Myh3, Myh8, Myh6, Myh13, and Myh14 isoform of MyHC in addition to the common isoforms like Myh7, Myh2, Myh4, and Myh1. The common isoforms of MyHC are usually found in the noncraniofacial muscle‐like limbs. Additionally, different isoforms of MyHC are expressed in a single muscle, for example, extraocular muscle expresses multiple MyHC isoforms.^62^ Another unique feature of the craniofacial skeletal muscle is related to the function of the satellite cells, adult muscle stem cells that are present under the muscle fiber lamina. Satellite cells are responsible for muscle tissue regeneration in case of trauma.^63^, ^64^ Craniofacial muscle cells and noncraniofacial muscle cells both contain the stem cells, but the noncraniofacial satellite muscle plays cells crucial role in the myogenic lineage differentiation by expressing a transcription factor called Pax7, where this transcription factor is not involved In the embryonic development of craniofacial muscle.^65^

Additionally, less PAX7 is expressed by the satellite cells of craniofacial muscles, but they still express a crucial transcription factor called Pitx2, which is essential for embryonic development.^66^ For example, the regenerative capacity of extraocular muscle is maintained irrespective of age and disease.^67^ Since satellite cells of craniofacial muscle are implicated in the regeneration of crucial facial muscles, they have the potential to become the vital target for craniofacial tissue regeneration.

In addition to this, recently, Michael R. Hicks confirmed that Human pluripotent stem cells could be induced to differentiate into skeletal muscle progenitor cells.^68^ Kim et al. successfully generated craniofacial myogenic progenitor cells from human induced pluripotent stem cells.^69^ The application of human pluripotent stem cells to create progenitor cells for skeletal tissue regeneration is valuable information to future research about its use in whole cranial tissue regeneration. Induce pluripotent stem cells derived craniofacial muscles could be used as an autologous source for craniofacial tissue engineering or reconstruction surgery.^70^ Further research is required to formulate induce pluripotent stem cell‐laden bioinks along with the factor that could induce the differentiation of the iPSC to skeletal muscle progenitor cells. At present, no standardize protocol is available for generating craniofacial myogenic precursor cells from human iPSCs. The immediate requirement is the standardization of such protocol by analyzing the crucial signaling pathways mechanisms during craniofacial embryonic myogenesis.^69^

## 6 CRANIOFACIAL TISSUE ENGINEERING

Although the ex vivo skeletal muscle tissue culture was developed around a century ago, the reconstruction of tissues from progenitors began in the early 60s of the last century when cross‐striated muscle cells were developed in the petri dish from chick embryonic muscle cells.^71^ The importance of extracellular materials for cell survival and proliferation was also pointed out by Konigsberg, which led to its widespread use in modern tissue engineering.^72^ Several materials of natural and synthetic origin (polycaprolactone‐based polymers, fiber, alginate) were identified and developed to fabricate the skeletal muscle tissues in the lab.

Further, to enhance the differentiation to the skeletal muscle, skeleton/scaffold of specific functionalities like support to the cell growth, mechanical strength, chemical, and electrical conductivity, soluble growth factors were developed. For more complex tissue engineering, which required more than one type of cell (e.g., tissues with the vasculature) co‐culture technique was developed, for example, skeletal muscle cells with fibroblasts to engineer the myotendinous junction or endothelial cells to vascularize muscle or with neural cells to obtain neuromuscular junctions.^73^ Despite the advance in skeletal muscle tissue engineering, the fabrication of fully functional skeletal muscle tissue is a distinct task. Specifically, the engineered skeletal muscle tissues are lacking in strength when matched with their natural equivalent.^74^ As identified by Konigsberg et al., researchers are now focusing on matching the ECM to mimic the microenvironment of the skeletal muscles. All the classical methods were limited to the 2D culture or co‐culturing; however, research has confirmed the role of the 3D framework composed of ECM and growth factors to facilitate tissue restructuring and engineering. The bioinks that facilitate the complex tissues' fabrication can be remolded to be useful for craniofacial tissue engineering. For this purpose, natural, synthetic polymers, decellularized ECM, or the composites of natural‐synthetic‐decullarized ECM can be used to prepare the iPSC or adult muscle cell progenitor laden bioinks.

Craniofacial tissue complex is involved in many critical functions, including mastication, speech, smile, and has high aesthetic importance.^75^ Craniofacial bones, skeletal muscles, ligaments, blood vessels, nerves complex, and teeth are the critical component of the craniofacial complex. Damage to the craniofacial complex could not only severely affect the overall functions of the face, but because of the aesthetic appeal, it could take a toll on psychosocial behavior. In a broad sense, damage to the craniofacial tissues has a physical and social impact, and hence accurate reconstruction to restore the functional and aesthetic appeal is urgently required.

During embryonic development, mesenchymal cells (MSc) originate from the neural crest, which then subsequently differentiate into almost all the craniofacial tissues, including bone, ligaments, tendons, cartilages, teeth, etc.^76^ The neural crest is the intermediate group of cells native to the vertebrates, originates from the ectoderm germ layer which later differentiates into smooth muscle, neurons, glia, melanocytes, and craniofacial bones and cartilages. For the formation of the craniofacial structure, MCs work with mesodermal cells.^77^ During the development of craniofacial tissues, MC bifurcates into two lineages, one into the terminally differentiate stage and the other linage give rise to the off‐spring mesenchymal cells.^78^ After complete morphological development of the craniofacial tissues, off‐spring MC continues to reside inside in all the cranial tissues as stem cells, which letter called as MC stem cells. In adults, MS stem cells help to maintain a constant turnover of the cells to keep the physiological function intact during injury; MC stem cells differentiate to regenerate the tissues of the craniofacial complex.^79^ Irrespective of the very high potential, the inherent natural ability of MC stem cells to differentiate or regenerate into craniofacial tissues is not yet studied. Hence, there is substantial scope to its utility in craniofacial tissue regeneration.

Despite the several advantages of the MS stem cells, craniofacial tissue generation turns out to be a difficult task because of the complexity of the craniofacial complex. It requires the combined effort of remotely similar disciplines like robotics, polymer chemistry, mechanical engineering, cell biology, genetics, and material science. In the engineered tissue, cells must know their place with respect to the other cells, must participate in the coordinated cell signaling pathways, and must differentiate and synthesize ECM. In this respect, craniofacial structures are very complex and offer several hurdles in their artificial tissue engineering. Initially, craniofacial tissue engineering was based on the principles of classical methods. The focus was on the isolation of the stem cells and using them for tissue engineering. Several human‐shaped craniofacial tissues, including bones and cartilages prototypes, were prepared using the MC stem cells. Adipose tissue was also fabricated from the MC stem cells to be used in facial tissue restructuring.^79^

Craniofacial muscles share several similar issues with noncraniofacial tissue engineering. This leads to use many of the tissue engineering concepts from noncraniofacial tissue engineering. But there are still enough differences that present unique hurdles, most of which are concerned with 3D orientation, complexity, and vascularization.

Apart from the congenital disabilities, the most common causes of craniofacial abnormality and damages are surgeries, trauma, cancers, sports injuries, etc.^80^ Out of these, craniomaxillofacial injuries contribute to major deformities, and congenital disabilities are the primary cause of concerns among the infants.^81^ In craniofacial muscle reconstruction, craniofacial bone plays a crucial role because they provide the anchoring platform for soft tissues and teeth. As craniofacial bones are the platform for the soft tissues of the craniofacial complex, accurate craniofacial bone reconstruction is essential to reinstate the regular functions of the complex.^82^ For example, Gaihre et al.^83^ addressed the defects of craniomaxillofacial bone by restoring it using biocompatible polymers like chitosan, alginate, cellulose, collagen, fibrin, and silk. The synthetic polymers used for scaffold preparation were poly (l‐lactic acid), poly(lactic‐co‐glycolic acid), polycaprolactone, and poly(propylene fumarate).^83^ Tu et al. managed to resolve the craniomaxillofacial bone defects by employing prosthesis composed of the hand‐made customized prosthesis of hydroxyapatite /epoxide acrylate maleic. After implantation, none of the patient has shown any complications.^84^ In a separate study conducted by Nunes et al. on nine patients with hydroxyapatite implants, bone ingrowth was observed with no indication of inflammatory reactions in the surrounding tissues.^85^ Goetz et al. used 3D printed scaffold made up of tricalcium phosphate to repair the defects of craniofacial bone.^86^ Rotaru et al. rehabilitate the craniofacial bone defects using customized 3D implants made up of autologous or alloplastic materials. In one radically revolutionary example, full‐face transplantation was done from a cadaver in 2005 in Italy.^87^ The full face transplant is reserved for the situation where the person has unrecoverable injuries to the face.^87^ In recent times, another important example of craniofacial restructuring using a complete full face transplant was conducted by a team of Spanish doctors.^88^ Furthermore, modern techniques like 3D printing, CT, and CAD/CAM have offered the possibility of the customized craniofacial implants made up of biocompatible material. The option of the customized implants has opened up a new arena in the field of craniofacial tissue engineering with personalized patient care. After 2005, countries like Spain, Italy, China, and the United States have effectively implemented the facial transplantation for the indications like traumatic injury, neurofibromatosis, and disfigurement.^89^, ^90^, ^91^, ^92^ Progress made in the craniofacial bone transplant and reconstruction is fundamental for the effective regeneration of facial soft tissues.

An alternative approach to rehabilitate craniofacial tissue damage is the use of prostheses. Conventionally prostheses used face several challenges; the most critical one is matching the prostheses appearance with the patients in terms of color, stiffness, size, and shape. Such matching is a tedious and time‐consuming process. 3D printing could help match the shape and size of the prostheses as per the patient's requirement, and the overall process is not labor‐intensive. Rehabilitation using prostates is commissioned only when surgical restructuring is not possible. A few of the major advantages of prosthetic rehabilitation is lower costs with shorter treatment time than surgical reconstruction.^93^ In craniofacial restructuring, typically, the prostheses are required to reconstruct the dental, oral, orbital, and nasal regions, and polysimethylsiloxane is the most widely used material for the fabrication.^94^ With the advance in 3D imaging and 3D printing techniques, the fabrication of such prostheses has changed considerably in terms of shape and time. At present, very limited prostheses fabricated using 3D printing technology used in clinical settings are available. However, the availability of advanced additive technology can be used in complex craniofacial engineering to enhance the quality and outcomes of prosthetic restoration. In the future, PDMS prosthetics printed directly using advance 3D printers could considerably improve the quality at a lower cost for the craniofacial application.

### 6.1 Craniofacial bone regeneration

Autogenous bone grafts, especially from the iliac crest and rib bones, are considered as the most trusted source for craniofacial bone regeneration.^95^ However, the origin of craniofacial and other long bone is from the different germ layers, which need to be considered for grafting. Another primary concern is similar to all the bone grafts, for example, inadequate supply and donor site morbidity. One critical post graft concern is the limited vascularization, which could lead to graft resorption and loss of structural features. Conservation of the periosteum layer and environment at the site of implantation was found to stimulate the revascularization process on the grafted bone.^96^ One major issue with the craniofacial bones is their complex 3D structure compared to the long bones, which make bones like iliac, fibula, or ribs difficult to restructure to fit into craniofacial bones morphology. To overcome the challenge of limited availability and complex 3D structure, tissue and bone engineering has promised the concrete approach to treat the defect of craniofacial bone defects by synchronizing active constituents, cells, and growth inducers.^97^ Craniomaxillofacial defects were successfully resolved in a clinical trial by using a stem cell‐mediated bone repair method. A similar approach was used to slow down the degeneration of bones in osteonecrosis of the femoral head and for prophylactic management of distal tibial fractures.^80^ The use of the stem cells for bone engineering is based on the original work carried out by Friedenstein et al., who reported the osteogenic differentiation from multipotent‐stromal‐cell and mesenchymal‐stem‐cell.^98^ Friedenstein was the first who reported that the bone marrow contains the specialized cells known as melanocytes, which are not only essential for the osteogenesis but also essential for the development of the native microenvironment.^98^ Due to their favorable osteogenic potential, MSC is considered an important cell source for facial bone tissue engineering.^99^ Adult bone marrow stem cells were the first to use for craniofacial bone regeneration among stem cells. Bone repair cells produced ex vivo also represent a class of stem cells potentially useful for stimulating osteogenic cell proliferation for bone regeneration.^100^ Ksigler et al. used bone repair cells successfully produced from the bone marrow cells to regenerate craniofacial bones.^101^ The experimental results confirmed the multilineage and clinical application of bone repair cells for craniofacial bone engineering.^100^

Although the association of fat cells with bone formation is an intense field of research, various studies indicate the counter relationship between the differentiation of bone marrow‐derived mesenchymal stem cells or stromal cells to the adipocyte and osteoblast lineage pathways.^102^ During the last decade, adipose‐derived mesenchymal cells appeared as a reliable source of cells for craniofacial bone tissue regeneration.^103^ One advantage of the adipose‐derived mesenchymal cells over the bone marrow cells is their scalability and easy access. Cowan et al. were the first who studied adipose‐derived mesenchymal cells for their osteogenic ability to manage the critical‐size mouse calvarial defects successfully.^104^ Very recently, in 2012, Gomes et al. treated the critical‐size calvarial defects with white adipose tissue. The recovery was confirmed with the expression of protein bone morphogenetic protein (BMP)‐2, which is essential for bone repair mechanism and homeostasis.^105^ Similarly, Azevedo‐Neto et al. transplanted subcutaneous adipose tissue to repair the craniofacial damage. Adipose tissue was found to stimulate craniofacial bone damage and confirmed by the expression of adipolactin (expression specific to adipose tissue).^106^ Craniofacial bone regeneration capacity of cells like amniotic epithelial cells, umbilical cord‐derived mesenchymal stem cells, and amniotic fluid mesenchymal cells was also evaluated. One major advantage of such cells is their ability to assist blood capillaries formation during bone healing.^107^

Craniofacial bone marrow cells were also studied for their bone regeneration capacity. In a recent rat study, mandible‐derived BMSCs showed higher bone mineralization as compared to the BMSCs. BMSCs from the marrow of mandibular or maxillary bones have shown better osteogenesis and stimulated higher expression of osteoblastic markers than the bone marrow extracted from the long bones of the same patients.^108^ On a similar line of research, BMSC obtained from the calvarial bones was found to stimulate bone regeneration.^109^ Despite the positive results obtained from the craniofacial bone marrow cells, limited availability is the major hurdle in its widespread application. In several studies, growth stimulators have been found to play a crucial role in stimulating the progenitor cells to osteogenesis. In recent times, the use of growth factors has received wider acceptance. One of the most commonly used growth stimulators used for craniofacial bone tissue engineering is the bone morphogenic protein.^110^

Despite the in vitro and ex‐in vivo success of craniofacial bone tissue engineering, its clinical application has several hurdles to overcome. This includes a limited supply of the autogenous progenitor cells, long‐term viability of the transplants, isolation, selection, storage of the stem cells, lack of proper 3D microenvironment for differentiation, and loss of multipotentiality character after six or seven cell cycles in vitro cell culture.^111^ One of the most potent remedies to such issues is the use of a 3D scaffold of ECM and seeding them with the progenitor cells or creating the 3D cell culture to match with the natural microenvironment. Table 1 summarized a few of the crucial applications of stem cells for craniofacial bone regeneration.

**TABLE 1:** Crucial applications of stem cells for craniofacial bone regeneration

S. no	Cells	Bone engineered	Remark	Objective	Reference
1	Bone marrow stromal cells (MSCs)	Cranial defects	Three‐dimensional computerized tomography (CT) scan revealed an almost complete repair of the defect of the experimental group at 18 weeks. This study may provide insight for the future clinical repair of cranial defect	The objective of this study was to investigate the potential of using autologous MSCs to repair cranial bone defects by a tissue‐engineering approach	112
2	Dental mesenchymal cell	Mandibular defects	This pilot study supports the feasibility of tissue‐engineering approaches for coordinated autologous tooth and mandible reconstruction, and provides a basis for future improvement of this technique for eventual clinical use in humans	Investigated simultaneous mandibular and tooth reconstruction using a Yucatan minipig model	113
3	Genetically engineered bone marrow‐derived mesenchymal stem cells overexpressing hypoxia‐inducible factor‐1α	Calvarial defects in rats	HIF‐1α‐overexpressing BMSCs dramatically improved the repair of critical‐sized calvarial defects, including increased bone volume, bone mineral density, blood vessel number, and blood vessel area in vivo	The hypothesis that HIF‐1α gene therapy could be used to promote the repair of critical‐sized bone defects	114
4	Bone marrow‐derived mesenchymal stem cells (BMSCs) genetically engineered transient expression of osteogenic/angiogenic factors and growth factor expression	Calvarial bone healing	BMSCs accelerated the bone remodeling and regenerated the bone through the natural intramembranous pathway	Augmented healing of critical‐size calvarial defects by baculovirus‐engineered MSCs that persistently express growth factors	115
5	Amniotic epithelial cells	Maxillary sinus	The obtained data suggest that scaffold integration and bone deposition are positively influenced by allotransplantated oAEC	The bone regenerative property of an emerging source of progenitor cells, the amniotic epithelial cells (AEC), loaded on a calcium‐phosphate synthetic bone substitute, made by direct rapid prototyping (rPT) technique, was evaluated in an animal study	107
6	Human umbilical cord mesenchymal stem cells	Rat cranial defects	hUCMSC and hBMSC groups generally had statistically similar bone mineral density, new bone amount and vessel density	hUCMSC and hBMSC seeding on macroporous calcium phosphate cement (CPC), and to compare their bone regeneration in critical‐sized cranial defects in rats	116
7	Amniotic fluid mesenchymal cells engineered on MgHA/collagen‐based scaffold	Sinus augmentation	The osteoinductive effect of a biomimetic commercial scaffold may be significantly improved by the presence of ovine AFMC	To evaluate whether commercial magnesium‐enriched hydroxyapatite (MgHA)/collagen‐based scaffold engineered with ovine amniotic fluid mesenchymal cells (oAFMC) could improve the bone regeneration process in vivo	117

### 6.2 3D printed craniofacial bones

Due to the various limitation of craniofacial bone tissue engineering, interest in 3D bioprinted craniofacial bone implants has grown considerably. 3D bioprinting is a relatively new field of research that involves the considerate use of scaffold materials, growth factors, and stem cells. The terms 3D printing and 3D bioprinting are often used interchangeably. Live cell printing often involves the use of inert scaffold or dense cell printing without scaffold. Several reports confirm that the 3D bioprinting can revolutionize the field of craniofacial bone tissue engineering. 3D bioprinted craniofacial bones offer several benefits to patients who require augmentation because of trauma or congenital flaws. CAD could help print the implants that match with the patient's anatomy, confirming excellent implant and bone contact.^118^ Bioprinting is still in the initial phase of development. One of the significant challenges in 3D bioprinting of craniofacial bone tissue engineering is developing and identifying suitable biocompatible materials that could support the function and growth of craniofacial tissues. Moreover, the materials must have proper crosslinking patterns to permit accurate deposition and bioactivity with sufficient strength over a long period of time.^34^

Embryonic cells create craniofacial and noncraniofacial bones. Somites lead the path to the axial skeleton, mesoderm from the limb bones, whereas the neural crest gives rise to the craniofacial bones and cartilages.^119^ Bone formation is the conversion of mesenchymal tissue to calcified bone, which is the target process needed to be translated in 3D bioprinting. The scaffold role is crucial in successful bone formation; hence, efforts are focused on developing feasible methods of fabrication. Traditional methods of bone tissue engineering like leaching, foaming, or frees drying lack the precision required to produce the accurate bone shape and size so that they could fit well in the puzzle of the craniofacial complex. On the other hand, in additive manufacturing controlled by a computer, the software program offers precise control over the topology and interconnectivity of the pores, which is crucial in the 3D bioprinting of craniofacial bones.

#### 6.2.1 Ceramics in 3D bioprinting of craniofacial bones

The present method available for handling craniofacial defects and damages is not reproducible and robust. It depends on the surgeon's skill and the natural response of the patient for the regeneration of the lost and damaged tissue. The 3D scaffold supports the growth of seeded cells at its offers the natural microenvironment. A technique like 3D bioprinting, which could design and construct the complex porous structure of scaffold, could revolutionize the field of craniofacial bone and tissue engineering. Components like plastics (synthetics and natural), ceramics, polymers, cells, and growth factors can be simultaneously used in the 3D printing of complex scaffolds for craniofacial bones. Such 3D printed scaffold with interconnected pores offers the advantage of fabricating patient‐specific implants, supporting cell growth and better vasculature formation.^120^

Ceramics are one of the most widely used materials for bio‐implants. Ceramics mainly used for its biological role in the human body are known as bioceramics. Bioceramics has several applications, including treatment, diagnosis and reinstall, and support of the bone function by creating its replica. They are basically of two types: (1) bioinert and (2) bioactive. Bioactive ceramics are biodegradable and promote the formation of new bone, whereas bioinert ceramics are implanted to interact actively with tissues through bonding. Bioinnert ceramic scaffold is mostly composed of alumina, alumina/borosilicate glass, alumina/SiC, etc., and Zirconia; on the other hand, bioactive scaffolds are mainly made up of mesoporous bioactive glasses13‐93, bioactive glass, 6P53Bglass, alkali‐free bioactive glass, hydroxyapatite, tricalcium phosphate, and calcium silicate.^121^

Bioinert ceramics are popular for implants because of their biocompatibility, corrosion resistance, and physiological stability. Alumina (Al_2_O_3_) and zirconia are the most widely used bioinert ceramics.^122^ Al_2_O_3_ was used first and is still popular because of its non‐toxic nature, durability over a long period, biocompatibility, and inertness toward the tissues.^123^ Zirconia is equally popular as Al_2_O_3_ because of its toughness among the available oxide ceramics.^124^ Other novel bioinert ceramics, which are gaining importance, include titanium dioxide (TiO_2_), silicon carbide (SiC), and carbon materials.^125^ For better mechanical strength, the incorporation of other material into Al_2_O_3_ or composites of hydroxyapatite was also proposed.^126^ For example, Glass/Alumina composite was used to make the complex structure using 3D printing technology.^126^ The CT scans and CAD created the prototypes of the complex structure, which is essential to disclose the minute details of the complicated craniofacial complex.^126^ To bring the unique properties of different material together, Gonçalves et al. combined hydroxyapatite, carbon nanotubes, and polycaprolactone to fabricate the 3D printed scaffold having interconnected pores for cell adhesion and growth.^127^

Hydroxyapatite and tricalcium phosphate are similar to natural bone and are considered attractive materials for bone tissue engineering. However, low mechanical barring strength and brittle nature limit the use of these materials in real clinical settings. Low strength is attributed to the calcium phosphate phases to which the HA decomposes. To enhance its mechanical strength, similar to alumina, HA is also combined with materials like zirconia, titania, calcium silicate, and alumina.^128^ HA forms a strong bond with bones, and alumina is stable biomaterial material but cannot interact with bones. Hence attempts were made to combine alumina and HA to form the bioinert but at the same time, bone and tissue interacting composite. In one of the approaches, AYDIN et al. fabricated the porous a‐TCP‐CeO_2_‐Al_2_O_3_ composite via using bovine bone‐derived HA and alumina ceramics.^129^ Several other reports also confirmed the role of calcium phosphate in bone reconstruction. The use of nanomaterials also offers an excellent method for bone tissue scaffold generation. Majid Rezaei et al. assessed the effect of nanoionization on biphasic calcium phosphate in the healing of canine mandible cavities.^130^

#### 6.2.2 Polymers and ceramic composites

Bioceramics have the excellent property of bioinnertness; however, the fragile nature of ceramics and the low mechanical strength of bioactive ceramics have restricted their clinical application. This makes it inevitable to pursue more research to be carried out to produce new materials or improve the properties of existing materials. Synthetic polymers have long been used for medical applications, including drug delivery and bone replacement.^131^ For example, poly(methyl methacrylate) (PMMA) and ultra‐high molecular weight polyethylene (UHMWPE) are widely used for hip replacement. Based on the experience and several studies, the polymers like polyethylene (PE), polypropylene (PP), polyurethane (PU), polytetrafluoroethylene (PTFE), poly(vinyl chloride) (PVC), polyamides(PA), PMMA, polyacetal, polycarbonate (PC), poly(−ethylene terephthalate) (PET), polyetheretherketone(PEEK), and polysulfone (PSU) are considered as the biocompatible polymers.^132^, ^133^ The latest interest in tissue engineering has renewed the research interest in biopolymers like poly(lactic acid) (PLA), poly(glycolic acid) (PGA), poly(ε‐caprolactone) (PCL), polyhydroxybutyrate.^134^ These polymers, either alone or combined with ceramics, could be used to fabricate the scaffold, seeded with different types of cells and growth factors. When combined with other materials, they are known as composites, and unlike the first‐generation classical materials, the composites are made for bio‐medical application, and hence they are termed as designer biomaterials or smart biomaterials.

Polymer‐like polylactic acid, which is linear aliphatic polyester, is also widely used for bone tissue engineering.^135^ Other than preserving its mechanical strength in physiological conditions, it is less viscous, biocompatible and its degradation products are non‐toxic. Vazquez‐Vazquez et al. used the air‐jet spinning technique to form a submicron coat of PLA over the 3D printed scaffold to analyze the cell (human fetal osteoblast cells) adhesion, cell‐material interaction, and proliferation.^136^ Polymers, like polycaprolactone and poly(lactic‐co‐glycolic acid) (PLGA), are widely popular in 3D bioprinting of bone tissue regeneration.^137^ These polymers are useful for their mechanical load‐bearing strength, but they do not support the bone growth of their own. This drawback can be potentially overcome by making the composite with calcium‐based ceramics (HA, calcium phosphate, tricalcium phosphate) and hydroxyapatite crystals. The addition of HA or tricalcium phosphate was found to enhance the mechanical strength of the polymers.^138^ Calcium phosphate and polymer‐like PLGA were found to stimulate osteoinductivity, an essential phenomenon to boost the osteogenesis process.^139^ While polymer–calcium ceramics composites have positively promoted bone regeneration in various animal models, the complete healing of the damage and defects is not reported. This prompted the research toward refining the design and materials for clinically viable implants. One such advancement in the design is the use of a bone ECM to support the scaffold bioactivity. Bone ECM could be decellularized or demineralized.^140^ For example, Bio Oss, developed by the Geistlich Biomaterials, is the natural substitute for bone and teeth implants. It is made up of the bovine trabecular bone from which most of the organic components are removed without changing the natural microstructure of the bone. A blend of decellularized bone matrix and polycaprolactone was compared with the Bio‐Oss, and no major difference in the differentiation of adipose‐derived mesenchymal stem cells into bone tissue was observed when cultured in hydrogels.^141^ Hung et al. in 2016 has confirmed that the decellularized bone matrix blend successfully healed the cranial defect in a mouse model.^142^ DMB, on the other hand, is the allogenic bone graft regularly used for filling the gaps and healing the defects. They are usually prepared by first removing the sift tissue, fats and blood followed by the acid‐based deminerilization and freez drying.^143^ The final product usually contains the collagen and BMPs and transforming growth factor‐beta 1, 2, and 3 granting it the osteogenesis properties.^143^ One limitation of demeralinized bone is its poor mechanical load‐bearing capacity, which has hampered its clinical development. One other challenge of such material is the limitation of processing it into a 3D porous scaffold to support the new vasculature formation and osteogenesis. This shortcoming was overcome by Freeman et al., who fabricated 3D bioprinted scaffold using composite PCL and decullarized bone filaments. The scaffold was also found to support angiogenesis and at the same time deliver the skeletal stem cells with growth factors to differentiate into osteoblasts.^144^

At the damaged bone site, osteoblasts, osteoclasts, and progenitor cells undergo order of sequence to restore the damaged part. This intrinsic ability of bone regeneration was coupled with the bone grafts for faster recovery. However, this classical method has several disadvantages, as discussed above (site morbidity, infection, immune activation in case of Xeno or allograft, limited supply in case of autografts, etc.). A few major requirements of the bone grafts are interconnected pores for oxygen and nutrient supply, attachment site for cell, support for proliferation and differentiation and tissue growth factors, high load‐bearing capacity. 3D bioprinting, along with the high precision over the structure, also overcomes the shortcomings of the classical methods of bone fabrications. Moncal et al. took over these issues and 3D bioprinted a bone tissue composed of poly(e‐caprolactone)/poly(d,l‐lactide‐co glycolide)/hydroxyapatite composite with interconnects micropores and high mechanical strength. The fabricated structure because the presence of mineralized bone tissue was found to support the bone regeneration via support to the angiogenesis process.^145^ Shah et al., in 2016, commercialized a new biomaterial compatible with 3d bioprinting used to fabricate hyperelastic bone. Hyperelastic bone consists of biodegradable 10% polymers used to link 90% of calcium phosphate ceramic. The hyperelastic bone has the ideal properties of bones like high porosity to support the bone growth, nutrient transport, and high mechanical strength.^146^

Like growth factors, ions like calcium play a crucial role in bone regeneration. Mg^2+^ ions also found to play an important role in bone metabolism. In recent time, role of several ions on bone tissue regeneration and engineering has been studied.^147^, ^148^ Odasa et al. recently fine‐tuned the release of such ions from the scaffolds composed of poly(lactic‐co‐glycolic acid)/Glass/ceramic composite and poly(lactic‐co‐glycolic acid)/Glass/ceramic composite.^149^ Mouse‐derived osteoblast‐like MC3T3‐E1 was found to reproduce well in the poly(lactic‐co‐glycolic acid)/Glass/ ceramic composite rather than the composite made up of without the glass particles.^149^

**FIGURE 4:**
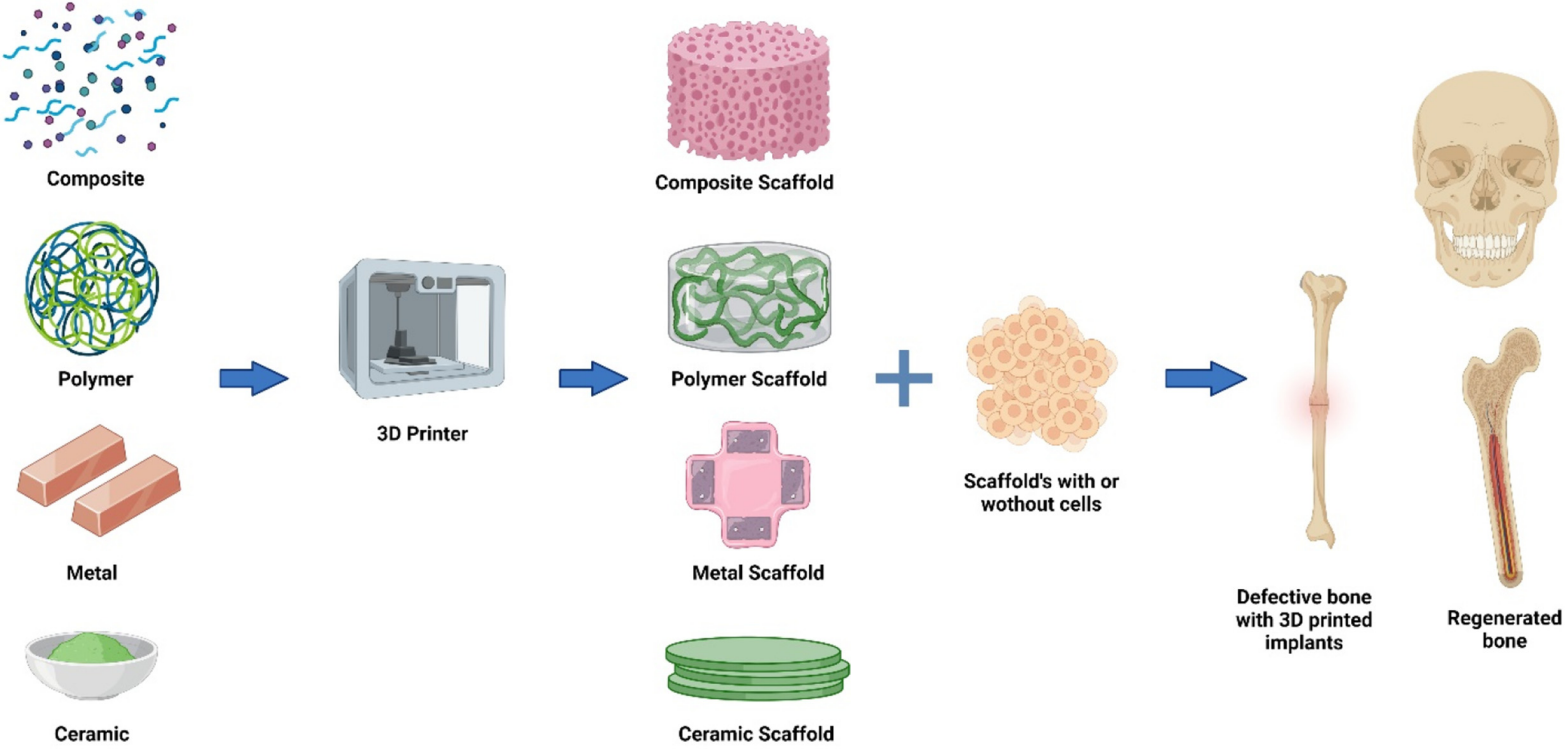
3D printing of the bone and bone substitutes

New polymer‐based material like carbohydrate polymer (HCCP) was used to repair the critical‐sized bone defects and compared with autologous bone and composite of poly(lactide‐co‐glycolide) with hyaluronic acid. Better bone density was observed in the group of animals implanted with carbohydrate polymer compared with poly(lactide‐co‐glycolide)/hyaluronic acid, and no difference was seen compared with autologous graft.^150^ The process of bone regeneration could be enhanced by using an anti‐osteoporosis agent like sodium alendronate. Sodium alendronate inhibits bone resorption; and hence, it was hypothesized that such agents could enhance the bone regeneration process. To check this hypothesis, Łucja Rumian et al. treated the craniofacial bone defect using a scaffold composed of titanium dioxide and poly(l‐lactide‐co‐glycolide) microparticles loaded with sodium alendronate. Sodium alendronate released from the scaffold was measured, and its usefulness in repairing critical size bone damage was confirmed.^151^ Figure 4 summarized the 3D printing of the bone and bone substitutes.

#### 6.2.3 Bone regeneration using gene‐activated matrices and the potential for bioprinting/3D printing

For guided, sustained, and controlled protein production, gene transfer to bone might be a promising therapeutic technique. Proteins, genes (viral and nonviral‐mediated delivery), and/or cells are sent to the bone defect location as part of tissue engineering procedures for bone repair. Furthermore, biomimetic scaffolds and scaffolds including bone anabolic drugs significantly improve bone healing.^152^ Sustained gene expression and continuous osteogenic protein synthesis in situ can be accomplished by implanting gene‐activated matrices (GAMs) in a way that supports osteogenesis and bone healing within osseous defects. Bone formation is accomplished through two major mechanisms. Due to a shortage of oxygen, endothelial cells proliferate and chondroblasts develop in the clot region, eventually leading in the creation of hyaline cartilage and intramembranous ossification where the preosteoblasts differentiate into osteoblasts, which in turn secrete ECM proteins and deposit calcium to harden the matrix.^153^, ^154^

Recombinant protein treatments to provide osteogenic cytokines and growth factors, delivery of nucleic acids (DNA, mRNA) encoding growth factors that stimulate bone formation, and transplantation of osteogenic cells at locations of bone abnormalities are all tissue‐engineering techniques for bone regeneration. Prior to implantation, osteogenic cells can be used as is or transduced using viruses or transfected with non‐viral vectors. These methods might be improved by integrating them with biomimetic biomaterial scaffolds to improve the therapeutic response and the bone‐repair process.^152^ Growth factor and cytokine protein‐based therapies are one option. Multiple growth factors, including osteoinductive and osteoconductive factors, have been found to stimulate angiogenesis and bone repair in a synergistic manner.^155^ The lack of selectivity for osteoblasts or bone‐forming cells in existing protein‐based treatments is a serious limitation. Proteins require high dosages (in milligrams) for direct clinical use due to their short half‐lives and fast disintegration, despite the fact that only tiny amounts are required for localized osteoinduction. In addition to the functional variability of proteins, these supra‐physiological dosages may cause undesirable consequences.^155^ The second method relies on the transplantation of critical cells that can manufacture the appropriate therapeutic growth factors at the implanted location. Ex vivo expanded autologous cells can be genetically modified to produce growth factors and then transplanted into the defect.^156^ Somatic cells, unlike stem cells, have limited potency, lack the potential to self‐renew, and are devoted to the creation of only one cell type. As a result, their application in complicated tissue engineering procedures is limited. MSCs that have been genetically engineered can be cultivated to generate a variety of growth factors, including bFGF and VEGF, BMP‐2 and BMP‐7, and VEGF and BMP‐4.^157^

Gene therapy, on the other hand, is thought to be an effective way of administering growth factors over time while avoiding the drawbacks of employing large protein concentrations. Multiple genes can be delivered at the same time, and tailoring is rather simple. pDNA encoding the desired therapeutic protein, a vector to allow cellular absorption of the pDNA, and in situ or ex vivo target tissue or cells that create the desired protein upon transfection are the three fundamental material components of gene therapy.^152^

The transgenes must be delivered selectively to the target cell nucleus to achieve effective transfection with low cytotoxicity and safety issues. Non‐viral gene delivery agents offer benefits over viral vectors in that they are less immunogenic, toxic, and have less pathogenic, carcinogenic, or other mutagenic problems than viral vectors, making them safer for clinical use.^158^

The in vivo gene delivery strategy, on the other hand, requires a high transfection efficiency of host cells to be successful. It is challenging to administer targeted gene delivery to certain cells using this method since the cells around the target tissue of interest may also be transfected. Ex vivo gene therapy allows for the selection, control, and examination of genetically changed cells prior to re‐implantation. GAMs are inert scaffold systems that incorporate viral or non‐viral gene delivery vectors and have been extensively studied and employed in wound healing and tissue engineering procedures.^159^ They act as 3D templates for tissue creation, and they can encapsulate and keep the gene within the sponge matrices for extended periods of time, which improves matrix deposition and blood vessel creation in growing bone tissue.^160^

It's worth noting that 3D bioprinting's uses are not restricted to organ printing. It also has a lot of potential in less‐explored areas including drug delivery via scaffolds, investigating disease causes, and developing tailored therapies.^161^ Bioprinting of rifampicin‐loaded PCL scaffold for possible osteomyelitis treatment,^162^ paracetamol‐containing PVA tablets with three different geometries,^163^ 5D additive manufacturing techniques to create personalized models of patients' pathology,^164^ and 3D bioprinting of GelMA‐based models to investigate the trophoblast cell invasion phenomenon, allowing studies of key placental functions.^165^ Rhodamine B was delivered using the 3D printed poly(ethylene glycol) dimethacrylate (PEGDMA) delivery system fabricated using a two‐photon polymerization.^166^

### 6.3 Craniofacial muscle engineering

Current treatment options for the damage to the craniofacial skeletal muscle tissues are the restructural surgery and transplantation of the autologous tissues, which sometimes could lead to the donor site morbidity. The modern approach includes the development of the scaffold to be seeded with the appropriate cell types to regenerate the skeletal muscles. This approach has the advantage of the restoration of the structural features and functions of the craniofacial complex and reduces donor site morbidity.

Much of the research in tissue engineering is focused on the skeletal muscles. In skeletal muscle tissue engineering, myoblasts obtained from the skeletal muscle itself are able to copy the process of muscle development.^167^ This process includes differentiation into myotubes. In skeletal muscle tissue engineering, a scaffold made up of biocompatible materials is important, and its interactions with the cells are crucial for tissue development and overall strength. The cells within the scaffolds should be able to produce their own growth factors and ECM. A biodegradable scaffold is made up of materials that initially support the tissue growth and letter degrade/substitute by the ECM produced by the seeded cells. This type of scaffold has no risk of immune activation and graft rejection. The most widely used biodegradable materials for skeletal muscle engineering are polyesters of naturally occurring α—hydroxy acids and collagen. Phosphate and SiO_2_ containing glasses have been available for some time now and have been shown to promote bone formation. Shah et al. have developed the phosphate‐based glass to be useful for craniofacial skeletal muscle engineering.^168^ The scaffold developed from the glass was found to release the non‐toxic ions while retaining its sustained degradation property. The glass was also tuned into the fibers, which offers a high surface area to volume ratio. This ensures more surface area for cell adhesion.^168^

Skeletal muscle cells are heavily influenced by surface topography, and several studies have confirmed the topographical effects on the cellular response.^169^ Among the structural features, parallel grooves are the pattern that is studied widely for skeletal muscle development and direction. In recent times, nanotopography has been the focus of research for its influence on skeletal muscle development and direction. Hydrogella is also widely studied to offer a similar environment for the 3D engineering of skeletal muscle. The primary research focus is on its ability to act as a topological surface to direct the skeletal tissue growth and allows the cell to adhere to it for growth and development. As discussed earlier, hydrogels could be prepare from the natural or synthetic polymers or the decellularized ECM. In skeletal muscle tissue engineering, myoblasts must migrate, align, and proliferate to develop the end structure. Fibrils are present in the hydrogel prepared from the ECM. Such fibrils, which are proteins, offer the cues to the cells to develop into the 3D structure.^170^ Landeret et al. used the same principle to develop the type 1 collagen hydrogel in which collagen fibers influence the myotube assembly in skeletal muscles.^171^ The hydrogel was also developed into the mold to guide the myoblast depending upon the shape and size. A scaffold made up of the composite of glass fibers in a collagen gel was also found to be useful for the differentiation of primary human masseter muscle‐derived cells.^172^ Similarly, photocrosslinkable acrylated gelatine was studied for its role in directing and aligning the myoblast in a 3D environment.^173^

Progenitor cells have the indigenous character to differentiate into the different cell types. Progenitor cells extracted from the craniofacial skeletal muscle have a very high potential of being differentiated into the respective muscle cells, making them the most useful cells to restore facial functions.^174^ In addition to the anatomical complications, the craniofacial skeletal muscle is different in origin from the other skeletal muscle; hence the proper selection of the progenitor cells is required, which must be from the facial muscle. In craniofacial tissue engineering, the progenitor cell is differentiated and expanded into the facial tissues.^174^ One of the well‐known progenitor cells with high myogenic differentiation potential is the mesenchymal stem cell. The characteristics like the formation of myotubes, development into muscle fibers, high proliferation, and synthesis of own ECM make them the most suitable progenitors for craniofacial skeletal muscle engineering.^175^ Mesenchymal stem cells (MSC) obtained from the bone marrow have been shown to have multilineage differentiation properties and hence are the suitable source for craniofacial muscle engineering. However, this source has the drawback of a deep invention surgical procedure and requires additional differentiation.^176^ Alternatively, skeletal muscle has its MSC known as satellite cells. The satellite has the proven myogenic differentiation property and hence is more suitable for skeletal muscle engineering as compared to the progenitor's cell obtained from the bone marrow.^176^ One added advantage of the satellite cells is its ability to migrate through the basal lamina sheets and differentiate to muscle cells immediately after the local trauma.^176^ Once differentiated to myoblast, they get easily attached to the preexisting cell of the damaged site.^175^ Facial satellite cells are also resistant to apoptosis, which makes them the most suitable cell type for tissue engineering.^177^

Craniofacial tissues are anatomically complex, and hence the scaffold required to tissue‐engineered such muscles is a design challenge. The scaffold must match the design requirement of the damaged tissue, and it must fit into the damaged part. Clinical imaging techniques like CT and MRI help to create the image of the size and shape of the scaffold to fit into the affected area. Mechanical stress, load bearding ability, and porosity are other crucial aspects of the scaffold that need to be considered during its design.^178^ 3D Scaffold is complex to fabricate. Hence the materials used to make it should have flexible physical and chemical properties.^179^ Direct or indirect solid‐free form fabrication is a widely used technique for the scaffold. Taboas et al. developed the hybrid of solid free form and classical sponge scaffold fabrication method. This method offers accurate maneuvering over the porosity and scaffold materials. The scaffold developed using this technique has porous architecture, and the method is adjustable with various polymers, ceramics, and composite materials.^180^ Direct solid‐free form fabrication involves layer by layer additive manufacturing, whereas indirect solid free form fabrication consists of casting the biomaterials into the fabricated mold.^181^ The most widely used biomaterials in both the techniques are PCL, polyglycolicacid(PGA),HA/TCPcomposites, polylactic/polyglycolic acid copolymers (PLGA),polycaprolactone (PCL), and polypropylene fumarate/tri‐calcium phosphate.

Irrespective of the techniques used for the scaffold fabrication, it must be capable of handling the bioresorbable and biodegradable materials and must produce the porous scaffold with large surface areas. One modern technique which has the potential to fabricate the complex scaffold is fused deposition modeling based on 3D printers. 3D printing and FDM offer the rapid fabrication of the porous scaffold and can copy the complex structure of natural tissues.^182^ The FDM technique is nearly the same as the solid free form process, with the addition of bioink extrusion heads that work on a platform working in all three directions to develop the 3D construct.^183^ Scaffolds composed of biodegradable materials are considered better than non‐biodegradable materials. Degradation of the scaffold after the tissue fabrication allows the simultaneous formation of ECM from the seeded cells, which further supports the cellular interactions and proliferation. However, the disadvantage of the degradable scaffold is that they are not easy to handle and are highly fragile. Irrespective of the drawbacks, natural biodegradable scaffolds allow higher cell adhesion when made with the fibrin and growth medium. When used together, thrombin and fibrin form the fibrin gel, replaced by the ECM proteins produced by the muscles progenitor cells in around 4 weeks.^184^ This is crucial because it is observed that the myoblast was found to grow faster in the degrading gels.^184^ Taken together, the ideal scaffold made up of the degradable or nondegradable polymers for craniofacial skeletal muscles can be fabricated by seeding the proginator cells into it.^185^

In craniofacial bone and soft‐tissue engineering, scaffolds are required to act as a template of the ECM and possess characters similar to the natural ECM. In addition, a major challenge in scaffolds engineering is its ability to form a bond with cells and support their proliferation. Previous reports have confirmed that the cell adhesion is directly linked with the surface area and, in turn, with the porosity. Interconnects pores channels are essential for the oxygen and nutrient movement to and fro. The scaffold should also possess mechanical strength matching with the natural tissues and organs and should degrade into non‐toxic metabolites, allowing natural ECM growth. Therefore, an ongoing need to identify novel scaffold platforms capable of facilitating bone engineering is required.

In the end, for craniofacial and non‐craniofacial bone and tissues, the major unmet challenge is vascularization. The cell is the structural and functional unit of the muscles and organs. The functional capacity of all organs and tissues depends on how well the cells are performing. For the natural metabolic functions of the cells, in the body, in almost every tissue and organ, cells are present at a distance of 200 micrometers from the blood vessel.^186^ Without blood vessels, cell survival is difficult as it provides oxygen and nutrients for metabolic functions. In classical tissue engineering, it is not easy to interconnect the vasculature of different layers. In this regard, 3D printing could help to solve this problem. Several reports demonstrate that the 3D printers could help fabricate the scaffolds that contain the vasculature that can compensate for the blood vessel functions.

## 7 OVERLOOK

Several natural and synthetic polymers have been investigated as a scaffold for bone tissue engineering, which includes collagen, chitosan, poly(caprolactones), poly(propylene fumarate), and polyesters such as polylactide, polyglycolide, and their copolymer poly(lactide‐co‐glycolide).^187^, ^188^ In addition to the polymers, ceramics and their composites have been used for craniofacial and non‐craniofacial bones. Tricalcium phosphate, which is a biodegradable derivative, has been widely used in research and clinical application.^189^ Tricalcium phosphate, due to high crystallinity, is more fragile, and its remolding features are different from the natural bone. The issue of crystallinity was overcome by developing the microsphere composite scaffold made up of PLAGA and low crystalline calcium phosphate. The scaffold was found to be highly porous, which is interconnected for cell migration, differentiation, and proliferation.^190^

Due to the recent progress in bone tissue engineering, the design, development, and manufacturing of porous materials for the scaffold to reinstall the natural state and function of the damaged parts have gained immense importance. Bioceramics and their composites have proven to be an immensely important material for bone scaffold development. 3D bioprinting technique in recent times has provided the advanced tool to process the complex designs of the scaffold‐like of craniofacial bones. Despite advancements in bioceramics research, its full potential is not yet exploited because of the few disadvantages. Several interesting innovations are taking place in ceramic chemistry. It was used to form the composite with the glass for tissue engineering. Glass composite offers better tissue interactions and assists bone development.^191^ An ideal scaffold stimulates bone regeneration. It should have a pore size sufficient enough for cell movement. Sol–gel derived glass is bioactive and has a porosity of nanoscale that can control the degradation rate.^192^

The development of multifunctional biomaterials from bioceramics with growth‐stimulating ions seems to be the feature of tissue engineering. In this regard, a composite of bioactive glass with ceramics and polymers has provided the sustained release of Sr, Cu, Zn, Ga, or Co, which stimulates the regeneration when released into the microenvironment.^193^

Overall, new and continuous advancement in design and development and novel process to fabricate the porous scaffold has added new dimensions to the utility of ceramics. 3D bioprinting has supplemented the development with specificity and complex bone fabrications. Further innovation in material science and engineering will contribute immensely to improving the lives of patients.

### 7.1 Specific challenges to the reconstructive cranioplasty

The field of tissue engineering is at the junction of bioengineering, material science, and medical science, which works to restore the functions of damaged tissues. It involves combining the live cells with a scaffold made up of synthetic or natural materials to build a fully functional 3D tissue. The tissue‐engineered structures are meant to be equal in function or better than the natural tissue. The four critical factors that govern the engineered tissue's success are: (1) scaffold, (2) type of cells, (3) ECM, and (4) growth regulators. Over the last three decades, the field of tissue engineering has registered exceptional progress. However, the development from 2D structures toward the 3D fully functional organs is still an unmet challenge. The best scaffold design, bioreactors, multipotent stem cells, standardized protocol, 3D bioprinting technology for microvasculature creation are under investigation to build functional tissues and organs. In the future, stem cells are the most critical unit for the success of functional tissue and organs. However, its application faces several significant, legal, and social challenges, which need to be addressed urgently.

Due to the rapid development in stem cell research, they hold the key to the success of tissue engineering. It was thought that adult stem cells have limited differentiation potential; however, recent evidence indicates that adult stem cells obtained from various tissue and organs have much better plasticity than previously thought. For example, muscle‐derived stem cells obtained from mice were found to differentiate into the hematopoietic lineage. Murine bone marrow stem cells when injected into a mouse with liver damage, new hepatocyte formation, and restoration of hepatic functions were observed.^194^

On the other hand, ESC derived from the 5‐day‐old embryos possesses the high potential of differentiation into various lineages. They possess the ability to differentiate into all their embryonic germ layers, and hence also known as pluripotent stem cells.^195^ After induction, they possess the unique ability to differentiate into the desired cell types. If induced with leukemia inhibitory factor, ESC can be preserved and expand indefinitely into a truly pluripotent phase. Human ESCs mostly differentiate into all types of somatic cells, the benefit of which is soft and hard tissue engineering is enormous.^196^ Irrespective of the advancement in stem cell research, identifying the required cell type for craniofacial bones and skeletal tissue is still a huge challenge. Other urgent challenges that are needed to resolve to involve (1) standardized induction protocol for the stem cell differentiation, (2) standardized adult stem cell isolation protocol, and (3) uniform and standard ethical approval protocol across all the cell lines.

Fabrication of thick functional tissue using classical tissue engineering methods, which generally has no provision of vasculature formation, is a significant challenge in developing thick functional organs.^197^ Appropriate microcapillaries and vessels could transport gas and nutrients to the proliferating cells and metabolic waste product back via veins for the excretion is the challenge. Capillary network for the functional tissues and organs is essential, and presently classical tissue engineering methods have no means to resolve the issue. It is essential to fabricate a vascular system of a minimum 10 μm diameter to support the growth of multilayer tissue of 2 to 3 mm^3^.^198^ At present, a 3D scaffold with inbuilt vasculature is the available design that could match the requirement of thick and hard tissue development. 3D printers are the tool available that could create the complex vasculature and scaffold for the same. In the near future, the foremost challenge is the development of the tissue microenvironment suitable for cell types essential for the optimum growth of the 3D vasculature.

Similar to the noncraniofacial tissues and bones, repairing the craniofacial tissue (hard and soft) requires a multidisciplinary approach. Craniofacial defects because of trauma, congenital, postcancer poses challenge of 3D scaffold, cell source, proliferation, stability, vasculature, etc. Transplantation of the autologous tissues and bone is considered as a gold standard remedy for the craniofacial tissues. However, the limited supply of such tissues has restricted their wider clinical use.^116^

Moreover, donor site morbidity like pain and infections demands a persistence search of the alternate approach and materials and methods for craniofacial bones and tissue repair. The ultimate goal of craniofacial reconstruction is to restore shape, size, functions, and esthetics with proper consideration of a change in anatomy in the case of younger patients whose structure is meant to change over the period.^199^ To date, rib bone, iliac rest, and scapula are considered as the suitable option in case of non‐availability of cranium bone.^200^ In general, an autologous transplant from the cranium is considered the gold standard because it can easily be integrated into the existing craniofacial skeleton. Furthermore, its resistance to the post‐surgical infection and adjustment to the growing skeleton offer added advantages. All these approaches are unsuitable if the damage is the considerably large and available quantity of autologous tissues is insufficient to fill the volume.

Over the last few decades, tissue engineering and 3D bioprinting have developed as a potential research field that could help fabricate the implants precisely similar to the lost part. As craniofacial tissues and bone are associated with various functions and esthetics, the implant of the same size and shape is crucial to retain all the viral characters. Craniofacial tissue engineering required the combination of accurate repair of bones and soft skeletal tissues. Often cranioplasty involves the regeneration of bones, vasculature, soft skeletal tissues, cartilage, skin, and mucosa, which require different types of cells to be cultured together. For example, the required stems cells, which differentiate into osteocytes and vasculature, are other and need to induce differently with different growth factors and conditions. Management of these requirements in classical tissue regeneration is difficult to achieve.

Use of growth‐regulating factors like BMP was also found to be useful in critical‐sized cranial bone defects. It stimulates the bone production and repair process and hence could play a crucial role in craniofacial bone tissue engineering.

The cell microenvironment plays a very important role in cellular signaling and proliferation. 3D structure of the ECM is essential for tissue regeneration. The 3D structure makes sure the smooth transport of the nutrient and oxygen through the vasculature. ECM not only provides the structural features to the tissues and organs but also makes sure the transport of the cell signaling molecules involved in cell differentiation and proliferation. Biocompatible materials used to prepare the scaffold / ECM should not only have sufficient mechanical strength, but at the same time, it should play the role of natural ECM. It is, therefore, a challenge to identify the biocompatible scaffold materials for craniofacial bone and soft tissue engineering.

## 8 CURRENT CHALLENGES IN CRANIOFACIAL BONE VASCULARIZATION

A mature vasculature can supply the oxygen and nutrients needed for cell survival, as well as secreted factors or proteins vital for ECM (ECM) synthesis. However, because of the structural specification and particularity of the developmental pattern of craniofacial bones, it is challenging to build a sophisticated neovascular system to nourish newly formed bones.^201^ The distance limit for cells to obtain efficient nutritional support and oxygen from the surrounding blood vessels is within 200 μm. However, bulk craniofacial bone defects are usually higher than 100‐μm or even 1‐cm thick because of trauma or tumor resection. In this case, transplanted or host‐recruited angiogenic or osteoblast cells may not survive long enough to ensure mature vessel ingrowth, resulting in compromised bone formation. Therefore, to prompt a thorough and reliable vasculature to support such bulk bone constructs in a short period is the main challenge in craniofacial revascularization. Furthermore, as efficient blood flow and blood pressure are necessary for oxygen and nutrients supplying the whole bone construct, the extent of anastomosis to host vasculature affects the ultimate functionalization of the newly formed vascular system. Thus, successful anastomosis with host vasculature is another issue that should be urgently addressed.

## 9 FUTURE DIRECTIONS

### 9.1 Exosomes in craniofacial tissue engineering and regeneration

Mesenchymal stem cells are a crucial cell component of craniofacial tissue engineering. Scaffold and growth factors assist the progenitor cell in adhesion, proliferation, and synthesize of their own ECM and signaling molecules. Additionally, MSCs can be extracted from the different tissues, including the craniofacial tissues, and can differentiate into different lineages, for example, bones, fats, muscle, nerves cartilages, etc. Unlike embryonic stem cells or induced pluripotent stem cells, MSCs use does not face ethical use issues. The mechanism involved in the differentiation of MSCs into different lineages is the topic of intense research. Theory suggests that after the transplantation, to induce regeneration and angiogenesis, MSCs may influence the other cells through paracrine function. The paracrine function of MSCs may be a crucial influencer for the success of the MSC‐based tissue engineering. Gnecchi et al. confirmed the paracrine role in tissue generation by injecting the intramyocardial injection of adult bone marrow‐derived stem cells to repair the myocardial tissue damage. The group proposed the mechanism for myocardial recovery, which involves the paracrine secretion of cytoprotective agents from the adult bone marrow‐derived stem cells.^202^ Lai et al. also confirmed the positive role of the paracrine influence of the exosome secreted by MSC in diminishing myocardial ischemia.^203^ The contents of exosomes are believed to influence the surrounding cells through paracrine function. Exosomes are found to contain the proteins which could act as a regulatory factor, miRNAs, and siRNAs. The most prominent content of exosomes is the miRNA that nonspecifically regulated the expression of several genes.^204^ Lai et al. confirmed that the MSCs contain around 150 miRNA, which could regulate the several crucial signaling pathways protein synthesis and may also involve in the repair and tissue regeneration.^204^ Since then, interest has been generated in using exosomes of the MSCs for craniofacial tissue engineering. Exosomes may exert the functions of stem cells that could influence the functions of different cells by regulating the ECM and growth factor synthesis, proliferation, migrations, and apoptosis. Exosomes may provide similar challenges and opportunities in craniofacial tissue regeneration.^205^

### 9.2 Delivering growth factors from the scaffold

A trend of incorporating the growth factors and signaling molecules inside the scaffold to induce the tissue regeneration and osteogenic and angiogenic differentiation of stem cells will be crucial to successful bone craniofacial tissue engineering. The osteoinductive growth factors like TGF‐B, BMP‐2,6,7,9, PDGF, VEGF, and FGF can be explored in craniofacial bone tissue engineering. Growth factors like transforming growth factor β (TGF‐β), fibroblast growth factor (FGF), vascular endothelial growth factor (VEGF), and platelet‐derived growth factor (PDGF) are known to stimulate bone regeneration.^206^, ^207^ Some of the BMPs and TGF β have been used in critical size craniofacial bone tissue engineering.^208^, ^209^ Although the complete mechanism is yet to be discovered, the reports to date indicate that the growth factors bind to the receptors present on the stem cells and induce them for osteoblastic differentiation via SMAD protein signaling pathway.^210^ The role of two isoforms of Bone morphogenic protein (BMP), BMP‐2 and BMP‐2, have been confirmed in bone healing, the presence of which in the graft has shown superior results.^211^ The clinical success of two commercially available bone grafts like Infuse® and Osigraft® are the two FDA‐approved collagen‐based scaffolds with BMP‐2 and BMP‐7 validates the crucial role of growth factors in craniofacial bone tissue. One another isoform, BMP‐9, which is a poorly studied growth factor, is also found to be potent in the differentiation of mesenchymal stem cells to bone cells.

Similarly, factors like FGF, PDGF, and HGF induce differentiation of the myogenic progenitor cells.^212^ Higher expression of proteins like myogenin and MHC and formation of larger myotubes were noted when satellite cells were induced by FGF. Double DNA synthesis was observed in the FGF‐treated cells, then the untreated cell. A similar effect of higher DNA synthesis and better satellite cell division was observed with the addition of PDGF.^213^ On the other hand, HGF was found to reduce the time for the onset of the cell cycle from 42–60 h to <24 h after isolation of satellite cells.^212^ These observations confirm the importance of restoring or conserving the native cell signaling in skeletal tissue engineering. These results indicate that if HGF is used in a combination of factors like FGF and PDGF. The tissue engineering process could be benefited from the early activation of cell division by HGF and enhance proliferation of the progenitor cells by FGF and PDGF. Similarly, when used with fibrin gel, IGF enhances myogenesis, and better initial proliferation was observed. This observation indicates its crucial role in skeletal muscle engineering. By using specific growth factors to induce satellite cell proliferation and differentiation, tissue engineers have efficiently fabricated skeletal muscle constructs.^214^ Because of the positive role of various growth factors in skeletal tissue and craniofacial bone tissue engineering, it is common to supplement the media and support system (scaffolds) with the appropriate combination action of growth factors.^214^

Loading of such growth factors in scaffold will ensure site‐specific delivery. Various successful attempts have been made to bring this concept to reality. Presently various biodegradable and nonbiodegradable polymers are used to prepare growth factors loaded scaffold. The methods to fabricate such scaffold are divided into two main types: (1) physical and chemical attachment of growth factors to the scaffold and (2) entrapment of growth factors inside the scaffold. The detailed discussion about the methods to load the growth factors on/in the scaffold is out of the scope of this review. However, it can be found in already published reviews.^215^

The addition of the growth factors could also negatively influence the mechanical load‐bearing capacity of the scaffold. Hence for future clinical application, critical evaluation is required to check the effect of the growth factors on the scaffold strength. Loading of the growth factors in the polymeric scaffolds has shown potential. This technique has proven to provide the mechanically sturdy scaffold, which could release the growth factors at the required site of action. Considerable research work is still required on the effect of growth factors and their action on the scaffold made up of polymers, composts, or ECM. In the future, standardized protocol is needed for the entrapment of the growth factors for the specific biomaterials used for scaffold fabrication. While fabricating such a system, is it necessary to find the polymers, composites, or combinations of biomaterials that could provide the maximum entrapment and provide continuous or sustained release of growth factors.

### 9.3 Hunting for the optimum scaffold

To ensure the natural microenvironment for the cell growth, tissue engineering commonly employs a 3D scaffold made up of biocompatible or biodegradable materials. 3D scaffold mimics the natural 3D microenvironment of the tissue where cell adheres to it, proliferate, produce ECM, and communicate with each other to work as a single unit.^216^ A highly porous scaffold ensures the success of cultured tissues and organs. For biomedical applications, the scaffold must be porous with the interconnecting network. Interconnect pore network ensures the movement of oxygen and nutrients, and waste materials. The porous surface of the scaffold not only offers higher surface area, which is essential for the cell adhesion, but also acts as a mechanical platform to the surrounding tissues and improves its mechanical stability.^217^ In addition to the 3D network structure, pores of the scaffold assist in guiding the new tissue formation. As discussed earlier, growth factors and specific ions essential for the cellular function are delivered via scaffold; porosity is such case helps to entrap more of such materials by offering higher surface area. The scaffold material could also be fine‐tuned to release such factors over a period of time, ensuring the sustained availability of growth factors. Porosity, although it has several advantages, the compromised mechanical property is also the function of highly porous scaffolds.^218^ Hence the optimum balance should exist between the mechanical and interconnected channels of the scaffold.

Conventional scaffold fabrication techniques like salt leaching, gas forming, phase separation, and freeze‐drying are not suitable for complex scaffold fabrication. Such techniques are often slow, require good fabrication skills, and do not offer precise control and reproducibility in fabrication. However, rapid prototyping techniques could obtain rapid fabrication of the complex scaffold.^219^ Conventional techniques also use toxic solvents that affect cell viability if not removed completely.^218^

Rapid prototyping fabrication techniques use 3D computer‐aided designing data, which enables fast fabrication with improving precision and mechanical properties.^220^ Rapid prototyping also offers precise control over the complex 3D architecture, design repeatability, and consistency.^221^ Although prototyping has several advantages, it is compatible with limited numbers of biomaterials as compared to the conventional tissue culture.^222^

Alternative approach is 3D printing, which involves placing continuous layers or droplets of biomaterials to form the 3D scaffolds. 3D printing offers high resolution and precise control over the pores as compared to the conventional methods. Various 3D printing techniques are discussed earlier in this review.^218^ Natural tissues like bones and skins have porous gradient systems in which porosity is not uniform throughout the structure; in fact, it is dispersed in such a way that enhances the complete performance of the structure.^223^ Porosity in the tissues like bones and skin increases at the center while it is minimum at the surface. Such gradient porosity enhances the mechanical strength and ensures cell migration nutrient movement, and facilitates waste removal. Dense porosity, on the other hand, other than the mechanical strength, provides better cell adhesion and signaling.^224^ Hence, in craniofacial bone tissue regeneration, a proper balance between mechanical strength and porosity, is required.^225^ For bone tissue engineering, the porosity of 20 to 1500 μm has been reported.^226^ In one of the reports, the pore size of 40 μm was found to be more populated than the 100 μm pores, which, however, was found to support the cell migration.^227^ In another study, the optimum cell migration and proliferation was found in the pore size of 300 μm.^228^ However, it should be noted that the cell proliferation and differentiation is also the function of the type of the cells, effects of growth factors, scaffold material, and overall conditions.^229^ One another consideration that is very crucial is angiogenesis. Scaffolds planted with the stem cells or endothelial cells along with VEGF and other growth factors have positively affected angiogenesis.^230^ Minimum porosity of 30–40 μm was found to support the growth of endothelial cells.^231^ The pore size of 160–270 μm was also found to support the process of angiogenesis.^232^ Fixed untunable scaffolds restrict the growth up to the size of scaffolds. The conventional scaffold made up of the nondegradable materials restricts the migration of cells and nutrients and can lead to deficiencies in the growing tissues. On the other hand, a fast degrading scaffold compromised the overall strength of the tissue and could risk the structural integrity of the construct before it could get strong enough to stand on its own. Overall, due to the immense importance of “smart” scaffolds, which could be autotuned with the cell differentiation, migration and strength are the future requirements.

### 9.4 Bone forms only within a vascularized site

Although much progress has been made in bone and skeletal muscle tissue engineering, however, lack of vascularization is holding back its clinical application. Hence, vascularization is the top research priority to translate the benefits of tissue engineering to the patients. The challenge is to fabricate the vascular network in the tissues before the transplantation, and it should integrate or anastomosis with the vessels of surrounding tissues. Moreover, vascular lumen fabricated either using the classical or 3D printed technology; it should have the lining of the endothelium to maintain the proper homeostasis.

The ultimate objective of vascularization is to provide the natural tissue environment and a means of transport of oxygen and nutrient to all the cells. Nutrient and oxygen play a crucial role in the proliferation and differentiation of the stem cell to the osteocytes and assist the restoration of the function of the target tissues. Imitating the exact physiological complexity of the tissues to develop the vessels is a major hurdle. Native tissue features like 3D microenvironment, ECM, cell–cell interactions, lymphatic system, and vascular network assist the nutrient movement and promotes tissue repair and regeneration naturally. Efforts are being made to copy these features of natural tissues to engineered transplantable implants. Several techniques, designs and biomaterials, and biological clues have to be used to develop the vasculature.

Recent reports suggest that in bone tissue engineering, bone formation takes place at the surface. In contrast, due to the limited supply of oxygen, its growth is limited at the center.^233^ It is noted that the osteocytes or their precursor cells are found to close to the blood vessels.^234^ To overcome the vascularization problem, Warnke et al. developed a titanium‐based scaffold of the mandible using a CT scan of the damaged part. The titanium scaffold was then first coated with hydroxyapatite and then with the human Bone Morphogenetic Protein‐7 (rh BMP‐7) and bone marrow‐derived mesenchymal stem cells. This scaffold was transplanted into the patient's own right latissimus dorsi muscle, allowing his own body to act as a natural bioreactor, which permitted heterotopic bone growth along with ingrowth of vessels from the thoracodorsal artery.^235^

Using the patient as a natural bioreactor to develop vasculature is close to nature. However, the concept of the formation of the vessels in vitro before the transplantation is emerging. This concept aims to create the functional vasculature inside the tissues ahead of the transplant. The vessels of such tissues should have the ability to the anastomosis with the preexisting vessels of the damage site ensuring, nutrient transport, differentiation, proliferation, and cell survival. The intrinsic benefit of the prevascularized tissues is their ability to maintain cell viability because of the preexisting vessels; this is not the case with the concept in which the body is used as a bioreactor to develop the vessels. Ex‐vivo generation of the vessels can engineer physiologically complex tissues with a better chance of survival.^236^ However, complete maturation and stabilization of the vessel before transplantation is the major hurdle in the clinical application of pervascularized tissues. Immature microvessels in the prevascularized tissues due to insufficient biological clues cause limited anastomosing with the host vessels. Such tissues also have a high tendency to undergo deterioration because of imperfect fusion.^237^ Immature microvessels are also more fragile, and higher gaps between the cells can cause oedema after the grafting.^238^ Present literature review proposes that the vasculature must be completely developed inside the tissues before transplantation. It also suggests the use of mural cells, stem cells, or mural cell precursors during the tissue engineering process. Mural cells play a critical role in the formation of mature vasculature. They are recruited by the endothelial cells via platelet‐derived growth factor B and vascular endothelial growth factor and play a crucial role in ECM production, which provides structural stability and growth factors essential for cell survival.

All the proof of concept studies, including 3D printed vasculature, have revealed the vessel's benefits in the success of tissue engineering. In the future, novel concepts are required to develop to facilitate the easy fabrication of vessels inside the thick tissues. Additionally, scale‐up studies need to be developed to successfully translate the proof of concept studies to the patients who are on the bed waiting for the transplants.

### 9.5 Creating interactions between tissue‐engineered skeletal muscle and the peripheral nervous system

To precisely recapitulate the physiological functions of natural tissues, the role of vasculature is crucial, but the interaction of tissues engineered skeletal muscle and vasculatures with the CNS via PNS will play a significant role in final integration. Skeletal muscles communicate with the CAN via motor neurons (alpha moto neurons, part of the somatic nervous system).^239^ To manage the functions of all tissues and organs, CNS integrates and analyze the sensory information received through the nerves. This coordination is accomplished through the connection between CNS and PNS. Principally, the connection between the tissues and nerve occurs via axons which are the fibrous projections of neurons. The neurons innervate the extrafusal muscle fibers of skeletal muscles, while the gamma motor neurons innervate the intrafusal muscle fibers of muscle spindles. The skeletal muscle of the head and neck is innervated by the alpha motor neurons, which are originated from the brainstem, whereas the remaining skeletal muscles of the body are innervated by the motor neurons originating from the spinal cord.

To effectively engineer the physiologically accurate skeletal muscle and bones of the craniofacial complex, it is crucial to engineering neuromuscular junction and the myotendinous junctions for effective coordination with the CNS. The myotendinous junctions are the unique region between the tendon and muscle where the actual transmission of force occurs.^240^ Few research groups focused on neuromuscular junctions in tissue engineering in the past. For example, Larkin et al. have developed 3D skeletal muscles having a myosin heavy chain capable of generating force. Further to enhance muscle contraction and develop adult myosin heavy chain isoforms, Larkin et al. cocultured fetal muscle cells with neural cells.^240^ Almost twofold better twitch and tetanus were observed in the muscles developed with a nerve than without the nerve explants when stimulated externally.^240^

As discussed earlier, myotendinous junctions are the specialized area where transmission of power takes place from muscle to the bones. To understand the mechanism of myotendinous junctions, Kostrominova et al. developed the 3D model of skeletal muscle—tendon construct. Myotendinous junctions in engineered tissues were developed by co‐culturing the self‐organized tendon constructs, or segments of adult or fetal rat tail was compared with in‐vivo junctions using the electron microscope.^241^

Neuropathological conditions, surgery, trauma can alter the innervation of the tissues, which could adversely affect the physiological functions of tissues and organs.^242^ Hence due to the significant importance of peripheral innervation in the normal pathological functioning of the organs, proper restoration and integration of the nerves is crucial. Provision of the innervation of 3D printed tissues and organs, which is meant to substitute the damaged part, will ensure the success of tissue engineering. In the future, it is essential to ensuring that tissue engineering will successfully face the challenge of innervation of engineered tissues using biomaterials and tissue engineering techniques. Although innervation is part of a complex set of challenges in tissue engineering, artificial tissues will significantly benefit from embedded neural cells that will ensure proper development and function.

## 10 CLINICAL APPLICATION OF THE CRANIOFACIAL TISSUE ENGINEERING

3D bioprinting technology is becoming increasingly important in medicine, with promising applications in bone restoration, rehabilitation, and regeneration, as well as expanding therapy choices in a variety of fields.^243^, ^244^, ^245^ It is a novel technology that poses a difficulty in both human and animal studies.

In terms of 3D printing in cranial bone regeneration, recent findings presented a comparison between human and animal investigations.^246^ Between 2021 and 2017, six human research were published, including two prospective clinical trials and four retrospective case reports.^247^, ^248^, ^249^, ^250^, ^251^ Studies included 81 patients (16 from the clinical trials and 62 from the case series). Mandibular bone abnormalities were the most often implanted 3D printed biomaterials, followed by calvarial, maxillary, and nasal deficiencies. The great majority of the abnormalities occurred as a result of tumor removal or trauma.^246^ For the length of observation, the immediate and long‐term bone regeneration was effective, and only one research reported one incidence of failure.^249^ Three investigations showed biomaterial infection and/or exposure, as well as fibrous invasion of the scaffold rather than bone penetration.^251^ However, these issues were effectively resolved, and the scaffold's long‐term viability was not jeopardized.

Reported research evaluated animal studies as well.^246^ The 36 animal studies were published during the period 2007–2017 and they included 614 animals where the most common used ones were rabbits, followed by rats, mice, pigs, sheep, and dogs. The majority of defects included calvarial, mandibular and maxillary. Histological, biochemical, histomorphometric, and microcomputed tomographic data in animal investigations showed that rapid and long‐term bone healing was successful during the time of observation. Some studies, however, only found bone growth around the scaffold structure, not on the interior. Only two studies revealed scaffold‐related problems.^252^, ^253^, ^254^, ^255^, ^256^, ^257^, ^258^ The human trials had a high rate of success with few major issues, but they were all found to have a high risk of bias, and the quality evaluation indicated that none of them met the criteria for a high‐quality research design. These research, on the other hand, are important discoveries for a cutting‐edge technology that is being used on humans. Animal studies, on the other hand, serve as a link between in vitro and in vivo research by highlighting key aspects of the histological and cellular backdrop of scaffold integration and bone regeneration.^246^ The use of 3D printing technology for tissue engineering has not yet gained widespread acceptance. 3D printing solutions for bone healing, particularly in the regeneration sector, appear to face several challenges. Surgical problems, material manipulation, and possible objections of the use of specific regenerative biomolecules in humans are all being addressed in ongoing study. On the assumption that numerous aspects should be taken into account to guarantee the effectiveness and widespread deployment of 3D printed bone scaffolding, the creation of 3D printed scaffolds should be viewed as a potential option for bone tissue restoration in craniofacial deficit (PMID: 30439546). Important factor represents collaboration between medical and engineering experts. Also, printing devices, in order to be faster and allow high resolution, should scale up. Finally, biomaterials research should create chemicals in the best possible combinations to provide the necessary functional, mechanical, and supporting qualities.^246^ Table 2 summarizes the current clinical and preclinical application of the 3D printing in craniofacial tissue engineering.

**TABLE 2:** Clinical and preclincal application of 3D bioprinting in craniofacial tissue engineering

S. no	3D printer	Clinical Indication and application	Material	Reference
1	Desktop M220, Apium Additive Technologies GmbH, Karlsruhe, Germany)	Cranioplasty/class III and class IV craniofacial defects	Polyetheretherketone	259
2	Custom 3D printer	Craniofacial osseous defects	Polymethyl methacrylate	260
3	Fused deposition using system Duplicator i3 (Wanhao, China)	Mandibular defects in an in vivo rodent model	LayFomm polymer (blend of polyvinyl alcohol and polyurethane)	261
4	Fused Deposition Modeling using commercial SpiderBot 4.0 HT 3D printer equipped with printing‐bed and internal chamber heating	In vitro study with potential bone tissue engineering applications	Polyetheretherketone nanocomposites with 10 wt% HA, SrHA and ZnHA	262
5	NA	In vitro analysis for craniofacial application	Titanium	263
6	3D‐powder printing system (Z‐Corporation, USA)	Vertical bone augmentation in the rabbit calvaria	Tri‐calcium phosphate	264
7	Additive manufacturing using customized 3D printer	Repeat surgery for restenosis	NA	265
8	Fused deposition modeling using Makerbot Replicator 2X, Makerbot, USA	Craniofacial bone reconstruction	15 wt% of zirconia (ZrO_2_) as well as 30, 35, and 40 wt% of beta‐tricalcium phosphate (β‐TCP) were compounded with polyamide 12	266
9	Selective laser sintering using EOSINT P800 printer (EOS GmbH, Krailling, Germany)	Critical‐sized mandibular defects in rabbits	Polyetherketoneketone	267
10	3DP Printer, T&R Biofab Co Ltd)	Nasal Septal Deformities	Polycaprolactone	268
11	Stereolithographic customized 3D printer	Mandible, including the temporomandibular joint	Polymethyl methacrylate	269
12	Extrusion‐based 3D plotting using Bioscaffolder 3.1, GeSiM mbH, Radeberg, German	Preclinical model study: Bone grafts for cleft alveolar osteoplasty	Calcium phosphate cement	270
13	Extrusion‐based custom 3D printer	Large cranial defects	Calcium phosphate and titanium	271
14	Custom 3D printer	Craniofacial Regeneration	Decellularized bone matrix particles combined with polycaprolactone	142
15	Custom 3D printer	Cranial implant	Polymethyl methacrylate	272
16	Objet500 Connex 3D and Stratasys	Cranioplasty	Polymethyl methacrylate	273
17	custom 3D printer	Mandibular avulsion injuries	Polyetheretherketone and titanium	274
18	Multichannel plotter of BioScaffolder 3.1, GeSiM, Radeberg, Germany	Cleft alveolar bone defects	Calcium phosphate cement and fibrin	275
19	Custom 3D printer	Rabbit calvarial and mandibular critical‐sized bone defects	Biphasic calcium phosphate powder (40 wt% hydroxyapatite ‐ 60 wt% β‐tricalcium phosphate)	276
20	Desktop Makerbot Replicator 2 (Makerbot Industries, Brooklyn, NY) and Stratasys Fortus 250mc (Stratasys, Edenprairie, MN).	Secondary orbital reconstructions clinical study	Polylactic acid	277
21	Custom 3D printer	Implant for rabbit calvaria	Polycaprolactone composite	278
22	Custom 3D printer	Critical‐size porcine craniofacial bone defects	Collagen‐polycaprolactone composite	279
23	WASP 4070 Industrial printer	Decompressive craniotomy requiring surgery for cranioplasty	Poly(methyl methacrylate)	280
24	Fused Deposition Modeling using V2‐B Dual Extruder; Kraftmaker, Taipei, Taiwan	Repair of alveolar bone defects in rats	Polylactide	281
25	Custom 3D printer	Rabbit nasal reconstruction	Polycaprolactone	282
26	Custom 3D printer	Nasal septal deformities	Polycaprolactone	283
27	MakerBot Replicator Desktop 3D Printer (5th Generation), MakerBot Industries, Brooklyn, NY)	Craniofacial reconstruction	Polylactic acid	284
28	Custom 3D printer	Clinical trial to evaluate the efficacy and safety of 3D printed bioceramic implants for the reconstruction of zygomatic bone defects	CaOSiO_2_‐P_2_O_5_‐B_2_O_3_ glass–ceramic	285
29	Custom‐made mechanical extrusion tool mounted on the Multi‐Arm BioPrinter	Calvarial bone regeneration	Composite made of polycaprolactone and poly (d,l‐lactide‐co‐glycolide)	286
30	Drop‐on‐demand 3D‐printer (3Z Studio, Solidscape, Multistation, Dinard)	Preclinical study for craniofacial bone repair	Calcium phosphate implants	287

## 11 CONCLUSION

Tissue engineering is a very complicated process, but this review presents an optimistic picture that with innovations in biomaterials, genetics, chemistry, and regenerative medicine, engineered tissues will have real clinical application in the coming days. In the days to come, 3D printing may make the process of tissue engineering more appealing. Looking at the present research, it seems that vascularization is the biggest hurdle that is holding back the trials of 3D‐printed organs and tissues. Considering the similarity of craniofacial bone and skeletal muscles with noncraniofacial tissue, the principles of later are applicable to craniofacial tissue engineering. However, it is crucial to remember that the craniofacial and noncraniofacial tissues do not only have the origin from the different germ layers, but they have different anatomical and physiological functions. Given the wide scope of tissue engineering, this review possibly could not cover all the technical facts that have contributed to the evolution of craniofacial tissue engineering. Hence the reader should refer to the recent in‐depth reviews and research articles covering the field of biomaterials, molecular biology, stem cells, and regenerative medicines.

## AUTHOR CONTRIBUTIONS

**Nitin Bharat Charbe:** Conceptualization (equal); writing – original draft (supporting); writing – review and editing (equal). **Murtaza Tambuwala:** Conceptualization (equal); writing – original draft (supporting); writing – review and editing (equal). **Sushesh Srivatsa Palakurthi:** Investigation (supporting); writing – original draft (supporting); writing – review and editing (equal). **Amol Warokar:** Writing – review and editing (supporting). **Altijana Hromić‐Jahjefendić:** Writing – review and editing (supporting). **Hamid Bakshi:** Writing – original draft (supporting); writing – review and editing (equal). **Flavia Zacconi:** Writing – original draft (supporting); writing – review and editing (equal). **Vijay Mishra:** Writing – original draft (supporting); writing – review and editing (equal). **Saurabh Khadse:** Writing – original draft (supporting); writing – review and editing (equal). **Alaa A. Aljabali:** Writing – original draft (supporting); writing – review and editing (equal). **Mohamed El‐Tanani:** Writing – original draft (supporting); writing – review and editing (equal). **Ãngel Serrano‐Aroca:** Writing – original draft (supporting); writing – review and editing (equal). **Srinath Palakurthi:** Writing – original draft (supporting); writing – review and editing (equal).

### PEER REVIEW

The peer review history for this article is available at https://publons.com/publon/10.1002/btm2.10333.

## Data Availability

Data sharing not applicable to this article as no datasets were generated or analysed during the current study
